# Neurodegenerative disease-associated microRNAs acting as signaling molecules modulate CNS neuron structure and viability

**DOI:** 10.1186/s12964-025-02199-8

**Published:** 2025-04-24

**Authors:** Victor Kumbol, Andranik Ivanov, Hugo McGurran, Jutta Schüler, Yuanyuan Zhai, Katarzyna Ludwik, Lukas Hinkelmann, Mariam Brehm, Christina Krüger, Judit Küchler, Thomas Wallach, Markus Höltje, Dieter Beule, Harald Stachelscheid, Seija Lehnardt

**Affiliations:** 1 Institute of Cell Biology and Neurobiology, Charité – Universitätsmedizin Berlin, Corporate Member of Freie Universität Berlin, Humboldt-Universität zu Berlin, and Berlin Institute of Health, Berlin, Germany; 2 Einstein Center for Neurosciences Berlin, Charité – Universitätsmedizin Berlin, Corporate Member of Freie Universität Berlin and Humboldt-Universität zu Berlin, and Berlin Institute of Health, Berlin, Germany; 3 Core Unit Bioinformatics, Berlin Institute of Health, Charité – Universitätsmedizin Berlin, Corporate Member of Freie Universität Berlin, Humboldt-Universität zu Berlin, and Berlin Institute of Health, Berlin, Germany; 4 Berlin Institute of Health at Charité – Universitätsmedizin Berlin, Core Unit pluripotent Stem Cells and Organoids, 13353 Berlin, Germany; 5 Institute of Integrative Neuroanatomy, Charité – Universitätsmedizin Berlin, Corporate Member of Freie Universität Berlin, Humboldt-Universität zu Berlin, and Berlin Institute of Health, Berlin, Germany; 6 Department of Neurology, Charité – Universitätsmedizin Berlin, Corporate Member of Freie Universität Berlin, Humboldt-Universität zu Berlin, and Berlin Institute of Health, Berlin, Germany

**Keywords:** Extracellular microRNA, Neuronal endocytosis, iPSC-derived human cortical neurons, Toll-like receptors, Neurodegeneration

## Abstract

**Background:**

Dysregulation of microRNA (miRNA) expression in the brain is a common feature of neurodegenerative diseases. Beyond their conventional role in regulating gene expression at the post-transcriptional level, certain miRNAs can act extracellularly as signaling molecules. Our study elucidates the identity of such miRNA species serving as ligands for membrane receptors expressed in central nervous system (CNS) neurons and the impact of such miRNAs on neurons in the context of neurodegenerative disease.

**Methods:**

We combined a machine learning approach with the analysis of disease-associated miRNA databases to predict Alzheimer’s disease (AD)-associated miRNAs as potential signaling molecules for single-stranded RNA-sensing Toll-like receptors (TLRs) 7 and 8. TLR-expressing HEK-Blue reporter cells, primary murine microglia, and human THP-1 macrophages were used to validate the AD miRNAs as ligands for human and mouse TLR7 and/or TLR8. Interaction between mouse cortical neurons and extracellularly applied AD miRNAs was analyzed by live cell imaging and confocal microscopy. Transcriptome changes in cortical neurons exposed to AD miRNAs were assessed by RNAseq and RT-qPCR. The extracellular AD miRNAs’ effects on CNS neuron structure were investigated in cell cultures of murine primary cortical neurons and iPSC-derived human cortical neurons by immunocytochemistry. We employed a mouse model of intrathecal injection to assess effects of AD miRNAs acting as signaling molecules on neurons in vivo.

**Results:**

We identified the AD-associated miRNAs miR-124-5p, miR-92a-1-5p, miR-9-5p, and miR-501-3p as novel endogenous ligands for TLR7 and/or TLR8. These miRNAs being extracellularly stable and active were taken up by murine cortical neurons via endocytosis and induced changes in neuronal inflammation-, proliferation-, and apoptosis-related gene expression. Exposure of both murine and human cortical neurons to the AD-associated miRNAs led to alterations of dendrite and axon structure, synapse protein expression, and cell viability in a sequence-dependent fashion. Extracellular introduction of the AD miRNAs into the cerebrospinal fluid of mice resulted in both changes in neuronal structure and synapses, and neuronal loss in the cerebral cortex. Most of the observed extracellular miRNA-induced effects on cortical neurons involved TLR7/8 signaling.

**Conclusion:**

Neurodegenerative disease-associated miRNAs in extracellular form act as signaling molecules for CNS neurons including human cortical neurons, thereby modulating their structure and viability.

**Supplementary Information:**

The online version contains supplementary material available at 10.1186/s12964-025-02199-8.

## Background

Neurodegenerative diseases, such as Alzheimer’s disease (AD), are characterized by progressive destruction of neurons. AD is marked by neuronal and synapse degeneration, which correlate with cognitive decline in both humans and animal AD models [1, 2]. Toll-like receptors (TLRs) contribute to such central nervous system (CNS) injury by triggering inflammation and cell-autonomous damage [3, 4]. The TLR family represents a class of membrane receptors, which mediates the host response to both pathogen-associated and host-derived molecular patterns [5]. TLR7 and TLR8 sense specific sequence motifs present in viral and endogenous single-stranded RNA (ssRNA), including microRNA (miRNA) [5, 6]. Both receptors are expressed in immune cells, such as microglia, but also in neurons [3, 4, 7, 8]. While proteins including α-synuclein and amyloid-β contribute to pathological processes in neurodegenerative diseases, such as Parkinson’s disease and AD, respectively [9, 10], the role of host-derived RNA herein is largely unresolved.

miRNAs are short (18–22 nt) non-coding RNAs that bind predominantly to the 3′ untranslated regions of mRNAs and regulate their expression post-transcriptionally [11]. MiRNAs are differentially expressed in neurodegenerative diseases [12–15]. Unlike other RNA species, they are extracellularly stable and circulate in body fluids, including plasma and cerebrospinal fluid (CSF), and therefore, are discussed as biomarkers for various diseases [16–18]. Moreover, miRNAs are transferred between cells and modify the recipient cell function implicating a potential role as extracellularly active signaling molecules [16, 19]. We have shown previously that neurons exposed to certain miRNAs undergo cell death through TLR7 [4, 20]. Yet, the miRNAs’ detailed mode of action on central nervous system (CNS) neurons and the consequences of an interaction between miRNAs and neuronal TLRs in the context of neurodegenerative disease are unresolved. Here, we investigated the effect of AD-associated miRNAs on cortical neurons, thereby characterizing the miRNAs’ function as signaling molecules in the CNS. By combining a machine-learning model with disease-linked miRNA databases and using a TLR reporter cell system for validation, we identified four miRNAs being dysregulated in AD, as TLR7/8 ligands. Live recording of murine cortical neurons revealed the uptake of extracellular miR-92a-1-5p and miR-124-5p, the kinetics underlying this endocytosis-based internalization, and the subcellular localization of the uptaken miRNAs. Extracellular AD-associated miRNAs induced alterations of murine dendrites, axons, and synapses, and triggered neuronal apoptosis, through TLR7 in vitro and in vivo. Transcriptome and RT-qPCR analysis of cortical neurons exposed to miR-92a-1-5p and miR-124-5p revealed changes in the gene expression pattern, particularly with respect to inflammation, proliferation-, and apoptosis-related signaling pathways. Exposure of iPSC-derived human cortical neurons to miR-9-5p and miR-501-3p preferentially activating human TLRs, led to alterations of neuronal structure, synapses, and cell viability in a sequence-dependent fashion via TLR7 and TLR8.

## Methods

### Mice

C57BL/6 and *Tlr7*^*−/−*^ mice were bred at the Research Institutes for Experimental Medicine (FEM), Charité – Universitätsmedizin Berlin, Germany. *Tlr7*^*−/−*^ mice were generously provided by S. Akira (Osaka University, Osaka, Japan) [21]. Mice were maintained and handled in accordance with the German Animal Welfare Act and the guidelines of the Charite Animal Welfare Committee. All animal procedures were approved by the Landesamt für Gesundheit und Soziales (LAGeSo) Berlin, Germany.

### Cell lines

HEK-Blue™ mTLR7, mTLR8, hTLR7, and hTLR8 cells and respective control Null2-k, Null1-v, Null1-k, and Null1 cells (Invivogen, San Diego, CA, USA) were cultured according to the manufacturer’s instructions. The growth medium consisted of Dulbecco’s Modified Eagle Medium (DMEM) (Invitrogen #41965062, Thermo Fisher Scientific, Waltham, MA, USA) supplemented with 10% heat-inactivated fetal bovine serum (FBS) (Gibco #10082–147, Thermo Fisher Scientific, Waltham, MA, USA), 100 U/mL penicillin/100 µg/mL streptomycin (1% P/S, Gibco #15140–122, Thermo Fisher Scientific, Waltham, MA, USA), 100 µg/ml zeocin (Invivogen #ant-zn-05, San Diego, CA, USA), and 10–30 µg/ml blasticidin (Invivogen #ant-bl-05, San Diego, CA, USA). THP-1 cells (E. Kowenz-Leutz, Max Delbrück Center for Molecular Medicine, Berlin, Germany) were cultured in RPMI-1640 medium (Gibco #21875-034, Thermo Fisher Scientific, Waltham, MA, USA), supplemented with 0.05 mM 2-mercaptoethanol, 10% FBS, and 1% P/S. THP-1 cells were differentiated into macrophages, as previously described [7]. In detail, cells were seeded into 96-well plates at 4 × 10^4^ cells/well and incubated for 48 h in media supplemented with 100 ng/mL phorbol-12-myristat-13-acetate (PMA; Merck #P8139, Darmstadt, Germany). Medium was replaced with fresh PMA-free medium for another 2 d before the experiments were initiated. All cells were maintained at 37 °C in humidified air with 5% CO_2_.

### Primary cultures of murine cortical neurons

Primary cultures of cortical neurons were prepared from forebrains of embryonic day (E) 17–18 mice, as previously described [4]. Briefly, meninges, superficial blood vessels, and cerebellum were removed from the cortices. Cortices were then homogenized and incubated with 0.5 mL Trypsin (0.5%) for 20 min at 37 °C. Trypsin reaction was stopped with 4 mL of 10% FBS. 100 µl DNase (Roche #10104459001, Basel, Switzerland) was added for 1 min and after several washing steps, the cell suspension was centrifuged at 300 rpm at 4 °C for 4 min. Supernatant was collected and centrifuged at 1200 rpm at 4 °C for a further 5 min. The pellet was resuspended in growth medium consisting of Neurobasal Medium (Gibco #21103–049, Thermo Fisher Scientific, Waltham, MA, USA) supplemented with 1% L-Glutamine (Gibco #25030–024, Thermo Fisher Scientific, Waltham, MA, USA), 1% P/S and 2% B27 supplement (Gibco #17504–044, Thermo Fisher Scientific, Waltham, MA, USA).

### iPSC-derived human neuron culture (iNeurons)

Human neural stem cells (NSCs) derived from the human induced pluripotent stem cell line BIHi005-A-24 that contains a stable integrated doxycycline inducible vector expressing NEUROG2 in the AAVS1 safe harbor locus were obtained from the BIH Core Unit pluripotent Stem Cells and Organoids, Berlin, Germany. NSCs were differentiated into human cortical neurons (iNeurons) by NEUROG2 overexpression, as previously described [22, 23]. In detail, neural stem cells were thawed and plated into 6-well plates coated with Geltrex (#A1413301, Thermo Fisher Scientific, Waltham, MA, USA) at a density of 1 × 10^6^ cells/well in Neural Expansion Medium (NEM) consisting of Neurobasal Medium/Advanced DMEM (#12491023) (1:1), 2% Neural Induction Supplement (#A1647801), and 1% penicillin/streptomycin (P/S) (all from Thermo Fisher Scientific, Waltham, MA, USA) supplemented with 3 µg/mL doxycycline (#D3447, Sigma-Aldrich, St. Louis, MO, USA) and 10 µM ROCK inhibitor Y-27,632 (#SEL-S1049-10MM, Selleck Chemicals, Houston, TX, USA). On the next day, medium was switched to NB-B27 Medium consisting of Neurobasal Medium, 1% B27, 1% P/S, 10 ng/mL NT-3 (#267-N3-025, R&D Systems, Minneapolis, MN, USA), 1X GlutaMAX (#A1647801, Thermo Fisher Scientific, Waltham, MA, USA), 10 ng/mL BDNF (#450-02-10, PeproTech, Cranbury, NJ, USA), and 200 ng/mL Laminin (#L2020, Sigma-Aldrich, St. Louis, MO, USA) with 3 µg/mL doxycycline. The medium was changed daily. On day 5, cells were detached with Accutase (#7920, Stem Cell Technologies, Vancouver, Canada) and replated onto Geltrex-coated PhenoPlate 96-well plates (#6055302, PerkinElmer, Shelton, CT, USA) at 3 × 10^4^ cells/well. The medium was changed daily until differentiation into mature iNeurons on day 14.

### Primary cultures of murine microglia

Primary cultures of microglia were prepared from mouse brains on postnatal day (P) 0 mice, as previously described [24]. In detail, meninges, superficial blood vessels, and cerebellum were separated from cortices. Cortices were then homogenized with 3 mL of Trypsin (2.5%; Gibco #15090–046, Thermo Fisher Scientific, Waltham, MA, USA) for 25 min at 37 °C. Trypsin reaction was stopped with FCS.100 µl DNase was added, and the cell suspension was centrifuged at 1200 rpm at 4 °C for 5 min. Pellets were resuspended in DMEM supplemented with 10% FCS and penicillin (100 U/ mL)/streptomycin (100 µg/mL), mechanically disassociated, and filtered through a 70-µm-cell strainer. Microglia were cultured in T75 flasks for 10–14 d in 12 mL DMEM at 37 °C in humidified air with 5% (v/v) CO_2_.

### Oligoribonucleotides and TLR agonists

Synthetic oligoribonucleotides were purchased from Integrated DNA Technologies (Coralville, IA, USA). The sequences of the synthesized oligoribonucleotides, with “Phos/” depicting 5′ phosphorylation, “*” depicting phosphorothioate bonds, and “Alex488N/” depicting Alexa Fluor 488 (NHS Ester) are as follows: mmu-miR-124-5p, 5′-Phos/C*G*U*G*U*U*C*A*C*A*G*C*G*G*A*C*C*U*U*G*A*U-3′; mmu-miR-92a-1-5p, 5′-Phos/A*G*G*U*U*G*G*G*A*U*U*U*G*U*C*G*C*A*A*U*G*C*U-3′; hsa-miR-9-5p, 5′-Phos/U*C*U*U*U*G*G*U*U*A*U*C*U*A*G*C*U*G*U*A*U*G*A-3′; hsa-miR-501-3p, 5′-Phos/A*A*U*G*C*A*C*C*C*G*G*G*C*A*A*G*G*A*U*U*C*U-3′; Alexa488-miR-124-5p, 5′Alex488N/C*G*U*G*U*U*C*A*C*A*G*C*G*G*A*C*C*U*U*G*A*U-3′; Alexa488-miR-92a-1-5p, 5′-Alexa488/A*G*G*U*U*G*G*G*A*U*U*U*G*U*C*G*C*A*A*U*G*C*U-3′; mutated control oligoribonucleotide (Mut.oligo), 5′-Phos/ U*G*A*G*G*U*A*G*A*A*G*G*A*U*A*U*A*A*G*G*A*U-3′. Loxoribine, R848, and TL8-506 were purchased from InvivoGen (San Diego, CA, USA). LPS was obtained from Enzo Life Sciences GmbH (#ALX-581-007-L002, Lörrach, Germany).

### HEK-Blue TLR reporter assay

HEK-Blue reporter and corresponding control cells were seeded at 5 × 10^4^ cells/well on 96-well plates, and one day later transfected with the oligoribonucleotides (all 10 µg/mL) using LyoVec (InvivoGen #lyec-1, San Diego, CA, USA) according to the manufacturer’s protocol. Cells were incubated with indicated treatments dissolved in HEK-Blue detection medium (InvivoGen #hb-det2, San Diego, CA, USA) for 24 h. The SEAP product was detected at 655 nm on a SpectraMax iD3 microplate reader (Molecular Devices, San Jose, CA, USA). For each experimental replicate, two wells were individually measured, and the average was used for evaluation.

### Enzyme-linked immunosorbent assay

Microglia isolated from C57BL/6 or *Tlr7*^*−/−*^ mice (3 × 10^4^ cells/well) or THP-1 human macrophages (4 × 10^4^ cells/well) were plated on 96-well plates. After overnight incubation, cells were stimulated with the oligoribonucleotides (10 µg/mL) complexed with LyoVec for 24 h. Thereafter, supernatants were collected and stored at − 80ºC. TNF concentrations in the supernatants were measured using the TNF Mouse or Human Uncoated ELISA Kit (Invitrogen, Waltham, MA, USA) according to the manufacturer’s protocol. For each experimental replicate, two wells were individually measured, and the average was used for evaluation.

### Quantification of extracellular miRNA uptake

Cortical neurons isolated from C57BL/6 mice were seeded on poly-D-lysine (PDL)-glass coverslips in 24-well plates at 2 × 10^5^ cells/well. After 3 d, cells were incubated with Alexa488-labelled miR-92a-1-5p, Alexa488-labelled miR-124-5p (both at 10 µg/mL), or Alexa Fluor 488 NHS-Ester (0.78 µg/mL, Thermo Fisher Scientific, #A20000, Waltham, MA, USA) and incubated at 37 °C for 1, 4, 8–24 h. Thereafter, cells were washed with phosphate-buffered saline (PBS), fixed with 4% paraformaldehyde (PFA) for 20 min, and nuclei were stained with DAPI. Soma and axons were immunolabelled with NeuN and Neurofilament antibody, respectively, mounted on glass slides, and imaged on a Nikon CSU-X1 Spinning Disk Confocal microscope (Nikon Corporation, Tokyo, Japan). Confocal image stacks (60X Nikon Plan Apo oil immersion objective, 1.4 NA, 0.5 μm step size) were acquired and subsequently analyzed in FiJi version-1.54 to quantify the fluorescence intensity of the uptaken Alexa488-labelled miRNAs or Alexa 488 Ester within the neurons. NeuN, DAPI, and Neurofilament-marked regions were obtained by thresholding the respective images and combined to generate an ROI of the total neuron area, and the Alexa488 fluorescence intensity within this ROI was measured.

### Live cell imaging of miRNA entry into neuronal endosomes

Primary murine neurons isolated from C57BL/6 mice were plated at 2 × 10^5^ cells/well in µ-Slide 8-Well Slides (Ibidi GmbH, #80826, Gräfelfing, Germany). After 3 d in vitro, culture medium was replaced with BrightCell NEUMO Photostable Medium (Merck Chemicals GmbH, #SCM146, Darmstadt, Germany) supplemented with 2% BrightCell SOS Neuronal Supplement (Merck Chemicals GmbH, #SCM147). On the next day, cells were incubated with a mixture of pHrodo Red Dextran (20 µg/mL Thermo Fisher Scientific, #P35368, Waltham, MA, USA) and Alexa488-labelled miR-92a-1-5p or miR-124-5p (10 µg/mL) or Alexa Fluor 488 NHS-Ester (0.78 µg/mL). 10 µl of Hoechst (Thermo Fisher Scientific, #R37605, Waltham, MA, USA) was added to stain the nuclei, and cells were immediately mounted for live cell imaging on a Nikon CSU-X1 Spinning Disk Confocal microscope. Confocal image stacks (100X Nikon Plan Apo oil immersion objective, 1.45 NA, 0.5 μm step size) were recorded at a frame interval of 2 min for 4 h recording sessions. The stage was maintained at 37 °C throughout the recording sessions. Live cell imaging data was analyzed using a custom macro in FiJi to quantify fluorescence intensity of pHrodo Red Dextran and Alexa488 within the endosomes.

### Analysis of neuronal endocytosis

Cortical neurons from C57BL/6 mice were seeded on PDL-coated glass coverslips in 24-well plates at 2 × 10^5^ cells/well. After 3 d in vitro, cells were treated with Dynasore (200 µM, Abcam #ab120192, Cambridge, UK) or DMSO for 1 h. Next, cells were incubated with a mixture of pHrodo Red Dextran (20 µg/mL) and Alexa488-labelled miR-92a-1-5p or miR-124-5p (10 µg/mL) at 37 °C for 4 h in the presence of Dynasore and DMSO. Cells were then stained with Hoechst, washed with PBS, fixed with 4% PFA for 20 min and mounted on glass slides. Cells were imaged on a Nikon CSU-X1 Spinning Disk Confocal microscope. Image stacks (0.5 μm step size) were acquired with a 100X objective. The average Z projections of the image stacks were created in FiJi. ROIs marking the endosomes, where Alexa488 and pHrodo Red Dextran co-localized, were generated, and the Alexa488 fluorescence intensity within these ROIs was measured. The fluorescence intensity of pHrodo Red within the entire image was also measured.

### Analysis of extracellular miRNAs’ effect on neuron structure and viability

#### Primary mouse neurons

Cortical neurons isolated from C57BL/6 or *Tlr7*^*−/−*^ mice (5 × 10^5^ cells/well) were cultured on PDL-coated glass coverslips in 24-well plates. After 3 d in vitro, cells were treated with oligoribonucleotides or TLR agonists, as indicated, for 5 d. Cells were washed and fixed with 4% PFA. Soma, dendrites, and axons were immunolabelled with NeuN, MAP-2, and Neurofilament antibody, respectively. Terminal deoxynucleotidyl transferase-mediated biotinylated UTP nick end labeling (TUNEL) was performed to label apoptotic cells using the In Situ Cell Death Detection Kit Fluorescein (#11684795910, Roche, Basel, Switzerland) following the manufacturer’s instructions. Using a fixed sampling strategy, six fields per coverslip were captured on an Olympus IX81 microscope (Olympus, Tokyo, Japan) with 40X objective. Images were analyzed using a custom macro in FiJi to quantify NeuN^+^ and TUNEL^+^ cell counts. NeuN^+^ cell counts were expressed as relative neuronal viability for each condition normalized to the control condition which was set to 100%. Apoptotic cells were expressed as the ratio of TUNEL^+^ to NeuN^+^ cell counts normalized to control. Neurite lengths were analyzed in FiJi using a macro adapted from MorphoNeuroNet [25]. Neurite degeneration analysis was performed as previously described [26] using a custom FiJi macro to generate masks of the dendrites and axons by thresholding the images, and measuring the total area of fragments and intact neurites. Particles between 0.2 and 25 µm^2^ were considered neurite fragments. The degeneration index was calculated as the ratio of the total area of neurite fragments to the total neurite area.

#### Human iNeurons

iNeurons (3 × 10^4^ cells/well) cultured in PhenoPlate 96-well plates were incubated with oligoribonucleotides or TLR agonists, as indicated, for 4 d. Oligoribonucleotides were complexed with LyoVec before application. For TLR inhibition experiments, cells were incubated with CU-CPT9a (50 µM, Invivogen #inh-cc9a, San Diego, CA, USA) or ODN 2087 (10 µM, Miltenyi Biotec #130-105-819, Bergisch Gladbach, Germany) for 3 h before and during the 4 d-incubation period. For caspase inhibition experiments, Z-VAD-FMK (100 µM, Sigma-Aldrich, #627610, St. Louis, MO, USA) was added 24 h before and during the 4 d-incubation period. Cells were washed and fixed with 4% PFA. Soma (NeuN), dendrites (MAP-2), axons (Neurofilament), general synapses (Synapsin), and excitatory synapses (VGLUT1) were immunolabelled with the respective antibodies. Apoptotic cells were labelled using the TUNEL apoptosis assay as described above. Cells were imaged (97 images/well) on an Opera Phenix High-Content Screening System (PerkinElmer, Shelton, CT, USA) with 40X objective. NeuN^+^ and TUNEL^+^ cell counts, and neurite lengths were analyzed automatically using Signals Image Artist (version 1.3.29, Revvity, Hopkinton, MA, USA). NeuN^+^ cell counts were expressed as relative neuronal viability for each condition normalized to the control condition, which was set to 100%. Apoptotic cells were expressed as the ratio of TUNEL^+^ to NeuN^+^ cell counts normalized to the control condition. For analysis of synaptic protein expression, images were processed in FiJi to generate ROIs where Synapsin or VGLUT1, and MAP2 were co-localized. The fluorescence intensity of Synapsin or VGLUT1 within these ROIs was measured. The neurite degeneration analysis was performed using the custom FiJi macro as described above for mouse neurons.

### siRNA transfection of iNeurons

On the penultimate day of differentiation (day 13), iPSC-derived human cortical neurons (iNeurons) were transfected with 1 µL TLR7 siRNA (10 µM stock, Silencer Invitrogen, #108895, Waltham, MA, USA) using 0.3 µL Lipofectamine RNAiMax (Thermo Fisher #13778075, Waltham, MA, USA) in 40 µL Opti-MEM I (Thermo Fisher #31985062, Waltham, MA, USA) per well on a 96-well plate according to the manufacturer’s protocol. The next day, fresh NB-B27 medium was added to the complexes, and cells were treated with oligoribonucleotides and TLR agonists as described above.

### Intrathecal injection of miRNA

Intrathecal injection of C57BL/6 and *Tlr7*^*−/−*^ mice was performed as described previously [7, 20]. Briefly, 10 µg of oligoribonucleotide dissolved in 40 µl of nuclease-free water was intrathecally injected into 6-8-week-old C57BL/6 or *Tlr7*^*−/−*^ mice, with 4–6 mice per experimental group. Control mice were injected with sterile PBS. After 72 h, mice were sacrificed and perfused transcardially with 4% PFA. Brains were removed and cryoprotected in 30% sucrose for 3 d. Subsequently, four coronal sections (Bregma − 1.94 mm, 0.14 mm, 1.54 mm, 2.80 mm) were prepared from each fixed brain. The slices were immunolabeled with anti-NeuN, anti-Synaptophysin, anti-MAP-2, and anti-Neurofilament antibodies, as described below. For quantification of neuronal loss, images were captured from twelve predefined fields in the cerebral cortex of corresponding brain sections across treatments using an Olympus BX51 microscope (Olympus, Tokyo, Japan) with a 60X objective. NeuN^+^ cells in each image were manually counted in a blinded fashion using the FiJi Cell Counter Plugin. For quantification of neurite length and synaptophysin abundance, images were captured from 16 predefined fields in the cerebral cortex of corresponding brain sections across treatments using an Olympus IX81 microscope (Olympus, Tokyo, Japan) at 64X magnification (40X objective with a 1.6X magnification changer). Images were subsequently analyzed in FiJi using a custom macro, as described above, to quantify the neurite length and degeneration index. Particles between 0.02 and 0.25 µm^2^ were considered neurite fragments. Synaptophysin abundance was quantified by measuring the mean synaptophysin fluorescence intensity across the entire image normalized to the number of cells.

### Immunocytochemistry and immunohistochemistry

Immunostaining was performed as described previously [20] using the following antibodies: NeuN (#ABN78), Neurofilament (#MAB5262), microtubule-associated protein 2 (MAP2, #MAB3418) (all obtained from Merck Millipore, Burlington, MA, USA); EEA1 (#ab206860) and Synaptophysin (#ab14692, both from Abcam, Cambridge, UK); TLR7 (#NBP2–27332) and TLR8 (#NBP2-24917, both from Novus Biologicals, Centennial, CO, USA); VGLUT1 (#135302) and Synapsin 1/2 (#106002, both from Synaptic Systems, Göttingen, Germany). Both primary and secondary antibodies (1:500) were applied in blocking buffer (PBS, 2% Normal Goat Serum, 0.2% TritonX-100).

### Bulk RNA sequencing

C57BL/6 cortical neurons were seeded at 2 × 10^6^ cells/well in 6-well plates. After oligoribonucleotide treatment total cellular RNA was isolated using the RNeasy Mini Kit (Qiagen, Hilden, Germany) following the manufacturer’s protocol. Briefly, cells were lysed and homogenized using QIAshredder spin columns (Qiagen, Hilden, Germany). One volume of 70% (v/v) ethanol was added to the homogenized lysate and transferred onto RNeasy spin columns. After a washing step, RNA preparations were treated with PureLink DNAse (Invitrogen #12185-010, Waltham, MA, USA) to remove contaminating DNA according to the manufacturer’s protocol. After several washing steps, RNA was eluted using 40 µL RNase-free water. Total RNA was stored at -80 °C until library preparation or cDNA synthesis. For sequencing, RNA quality was assessed using an Agilent RNA ScreenTape (Agilent Technologies, Santa Clara, CA, USA). mRNA sequencing libraries were prepared using the Illumina TruSeq Stranded mRNA kit (Illumina, San Diego, CA, USA) according to manufacturer’s instructions. Sample libraries were multiplexed and sequenced on a NextSeq 500 platform (Illumina, San Diego, CA, USA) using 75 bp single-end sequencing. 29–71 million reads per sample were generated.

### Bioinformatic analysis

Bulk RNAseq reads were mapped to the mouse genome (GRCm38, version M12 (Ensembl 87)) with STAR version-2.7.3a [27]. Reads were assigned to genes using FeatureCounts SUBREAD version-v2.0.0 [28] with the following parameters: -t exon -g gene_id -s 2. For the differential expression analysis, we used DESeq2 version-1.32.0 [29] with default parameters. We filtered genes that had less than 5 counts in at least 3 samples. Upregulated and downregulated genes were subjected to gene set enrichment analysis (GSEA) with R/tmod package version-0.50 [30] against the MsigDB Hallmark gene set collection. Significance of enrichment was assessed using the hypergeometric test (tmodHGtest).

### Real-time PCR

Total cellular RNA from treated neurons and microglia was isolated using the RNeasy Mini Kit (Qiagen, Hilden, Germany) following the manufacturer’s protocol. For cDNA synthesis, 1 µg of total RNA from was used in a 25 µL reaction volume with the following reagents according to manufacturers’ protocol: M-MLV Reverse Transcriptase 5X Reaction Buffer (#M531A, Promega, Madison, WI, USA), M-MLV RT (#M170A, Promega, Madison, WI, USA), Oligo(dT)18 Primer (#S0131, Thermo Scientific, Waltham, MA, USA), dNTP Mix (#18427013, Invitrogen, Waltham, MA, USA), Rnasin Ribonuclease Inhibitor (# N2511, Promega, Madison, WI, USA), and RNase-free water. All qPCR assays were performed on a StepOnePlus Real-Time PCR System (Applied Biosystems, Waltham, MA, USA) in 25 µl reaction volumes containing 1 µl of template, 300 nM of each primer, 1×SYBR Green PCR Mastermix (#4309155, Applied Biosystems, Waltham, MA, USA) according to manufacturer’s instructions. All conditions were performed in triplicate, and the average Ct value was used. Expression data are expressed as 2^–∆∆Ct^ normalized to Actin. Primer sequences were designed in house or obtained from the PrimerBank database [31] and commercially synthesized by Integrated DNA Technologies (Coralville, IA, USA). Primer sequences are available in Additional file 1: Table S1.

### Western blotting

Primary neurons were homogenized in 250 µl RIPA buffer (#R0278) containing 1:100 protease inhibitor (#P8340) and 1:100 phosphatase inhibitor (#P2850), all from Sigma-Aldrich, Missouri, MO, USA. Pierce BCA Protein Assay Kit (Thermo Fisher Scientific, #23227) was used to determine the protein concentration according to manufacturer’s instructions. 40 µg of total protein were resolved on NuPAGE Bis-Tris gels (Thermo Fisher Scientific, #NP0322PK2, Waltham, MA, USA) and transferred onto a nitrocellulose membrane using the iBlot2 Transfer device (Thermo Fisher Scientific, Waltham, MA, USA). Membranes were imaged on an Azure 400 Imager (Azure Biosystems, Dublin, CA, USA) after incubation of primary antibodies and secondary antibodies according to the manufacturer’s protocol: cleaved caspase-3 (1:1000, Cell Signaling Technology, #9661, Danvers, MA, USA); anti-beta actin (0.5 µg/mL, Sigma-Aldrich, #A1978-100UL, St. Louis, MO, USA); donkey anti-rabbit IgG-HRP (#sc-2313); goat-anti-mouse IgG-HRP (#sc-2005) both from Santa Cruz Biotechnology (Dallas, TX, USA). The images were exported as TIFFs and the relative band intensities were quantified using FiJi.

### Statistical analysis

Data are expressed as indicated in the figure legends. Statistical differences over all groups were analyzed with Kruskal-Wallis test followed with Dunn’s multiple comparison tests. Statistical differences between two specific groups were analyzed with the parametric two-tailed Student’s *t*-test or non-parametric Mann–Whitney test. The Mann–Whitney test was used instead of the Student’s *t*-test if the data failed the Brown-Forsythe test for homoscedasticity. For neurite length and degeneration index data, outliers were identified and excluded using the ROUT method (Q = 0.1%) in GraphPad Prism before analysis. Differences were considered statistically significant when *P* < 0.05. GraphPad Prism version 9.50 for Windows (GraphPad Software, San Diego, CA, USA) was used to perform all statistical analyses.

## Results

### AD-associated miR-124-5p, miR-92a-1-5p, miR-9-5p, and miR-501-3p are endogenous ligands of mouse and/or human TLR7/8

**Fig. 1:**
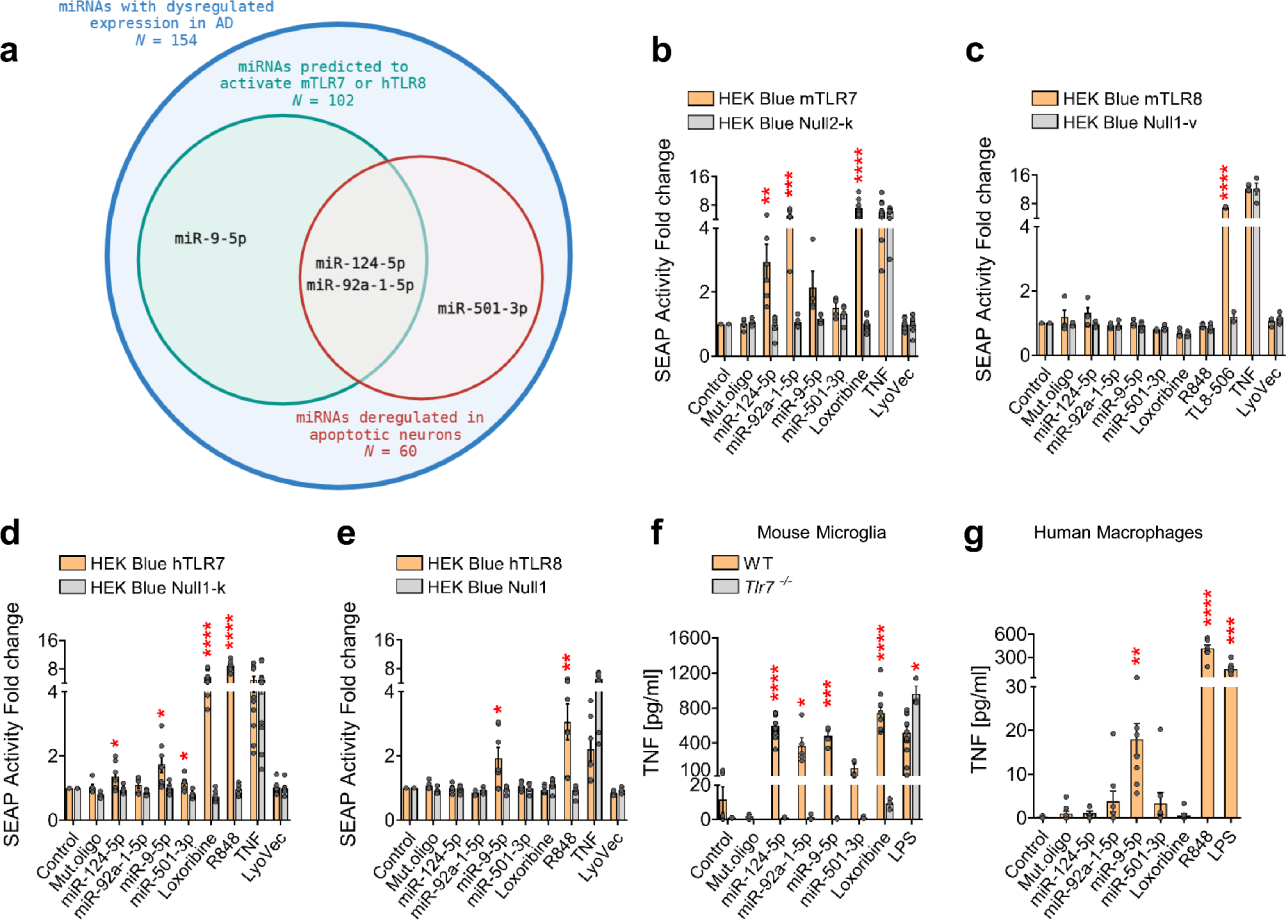
AD-associated miRNAs directly activate TLR7 and/or TLR8. (**a**) Selection of miRNAs dysregulated in AD, acting as potential signalling molecules for TLR7/8. HEK-Blue cells co-expressing (**b**) mTLR7, (**c**) mTLR8, (**d**) hTLR7, or (**e**) hTLR8, and an NF-κB/AP1-inducible secreted embryonic alkaline phosphatase (SEAP) reporter gene, were incubated with 10 µg/mL miRNA, as indicated, or mutant oligoribonucleotide (Mut.oligo) for 24 h. R848 (100 ng/mL), loxoribine (1 mM), TL8-506 (10 µg/mL), and TNF (100 ng/mL) served as positive control. Parental Null2-k, Null1-v, Null1-k, and Null1 cells and LyoVec served as negative control. Bars represent the mean ± SEM (*n* = 4–8). *P < 0.05; **P < 0.01; ***P < 0.001; ****P < 0.0001; unpaired *t*-test, compared to the respective parental HEK cells. (**f**) Microglia from C57BL/6 (wild-type, WT, *n* = 4–8) or *Tlr7*^*−/−*^ (*n* = 3) mice and (**g**) THP-1 macrophages (*n* = 6–8) were incubated with 10 µg/mL of indicated miRNAs or Mut.oligo for 24 h followed by TNF ELISA. Unstimulated cells served as negative control. LPS (100 ng/mL), loxoribine (1 mM), and R848 (100 ng/mL) served as positive control. Bars represent mean ± SEM. *P < 0.05; **P < 0.01; ***P < 0.001; ****P < 0.0001 (**f**) compared to the corresponding *Tlr7*^*−/−*^ group; unpaired *t*-test; (**g**) compared to control; Kruskal-Wallis test with Dunn’s post-hoc analysis

We sought to decipher the effects of extracellular miRNAs acting as signaling molecules on cortical neurons in the context of neurodegeneration. To select suitable miRNA candidates for this study, we used BrainDead, a structure-aware machine-learning algorithm, which predicts small RNAs as potential TLR7/8 activators [32]. Although originally trained with data derived from assays using mouse microglia, BrainDead can predict RNAs acting as ligands of human TLRs [32]. Considering published data derived from assays testing the activation of human embryonic kidney (HEK) human TLR8 (hTLR8) reporter cells by miRNAs [20, 33], the BrainDead algorithm was finetuned to predict small RNAs specifically activating this receptor. One hundred and fifty-four miRNAs annotated as being dysregulated (i.e. increased or decreased expression) in AD in the PhenomiR v2.0 database [34] were analyzed by both the original and finetuned BrainDead algorithm. Out of the miRNAs with dysregulated expression in AD, 102 miRNAs were predicted to activate mouse TLR7 (mTLR7) or hTLR8 (Fig. 1a). Out of the miRNAs with dysregulated expression in AD a second group of 60 candidate miRNAs was identified by re-analyzing previously published small RNA sequencing and microarray data derived from apoptotic cortical neurons releasing miRNAs [20, 33]. Out of these two identified subgroups we finally selected four miRNAs, namely miR-124-5p, miR-92a-1-5p, miR-9-5p, and miR-501-3p for further investigation (Table 1), taking into account distinct features of the selected miRNAs, as follows. miR-124-5p and miR-9-5p, abundantly expressed in mouse and human brain and both comprising sequences conserved in mouse and human, represent brain-specific miRNAs [35–38]. While miR-124-5p expression in AD brain is reduced [35], miR-9-5p expression is increased in brain tissue and CSF of AD patients [39, 40]. miR-92a-1-5p and miR-501-3p are also expressed in mouse and human brain [36, 38], and the sequences of mouse and human miR-92a-1-5p and miR-501-3p differ by 3 and 2 nucleotides, respectively. miR-92a-1-5p expression in AD brain is enhanced, while miR-501-3p expression is increased in both brain and serum of AD patients [41–43].

Next, the AD-associated miRNAs selected above were tested regarding their potential to activate TLR7/8 using HEK reporter cells overexpressing either mouse or human TLR7 or TLR8. As a control for miRNA sequence specificity, an oligoribonucleotide featuring a mutated sequence of the miRNA *let-7b* (referred to as Mut.oligo thereafter), which does not activate TLR7 [4], was used. While miR-124-5p activated both human and mouse TLR7, miR-92a-1-5p exclusively activated mTLR7 (Fig. 1b, d). miR-9-5p activated hTLR7 and hTLR8, whereas miR-501-3p activated hTLR7 (Fig. 1d, e). None of the miRNAs activated mTLR8 (Fig. 1c). Mut.oligo activated neither mouse nor human TLR7/8, as expected [4]. Loxoribine, a synthetic TLR7 agonist, activated mTLR7 and hTLR7, while R848, a TLR7/8 agonist, activated hTLR7 and hTLR8, and TL8-506, a TLR8-specific agonist, activated mTLR8, as expected (Fig. 1b-e).

To confirm the functional relevance of TLR activation by extracellular AD-associated miRNAs in immune cells, primary murine microglia, which express TLR7 and TLR8 [44], were exposed to miR-92a-1-5p, miR-124-5p, miR-9-5p, and miR-501-3p. Out of these, miR-92a-1-5p, miR-124-5p, and miR-9-5p induced TNF release from microglia (Fig. 1f). This response required TLR7, as cytokine release was abolished in *Tlr7*^*−/−*^ microglia (Fig. 1f). The miRNA-induced microglial cytokine response was in line with the respective miRNA’s ability to activate mTLR7 (see Fig. 1b). In contrast, miR-501-3p, which did not activate mTLR7 (see Fig. 1b), failed to elicit cytokine release from microglia (Fig. 1f). Also, human macrophages were tested. Although weakly (i.e. TNF concentration peak at 20 pg/mL), human THP-1-derived macrophages responded to extracellular miR-9-5p (Fig. 1g). However, miR-92a-1-5p, miR-124-5p, and miR-501-3p did not induce such a response, mirroring the results derived from hTLR8 reporter cells, which were exclusively activated by miR-9-5p (see Fig. 1e).

In summary, extracellular miR-124-5p activated mTLR7 and hTLR7, while miR-92a-1-5p exclusively activated mTLR7. miR-9-5p activated hTLR7/8, while miR-501-3p activated hTLR7 only. Except miR-501-3p, the tested miRNAs induced cytokine release from mouse microglia and/or human macrophages. Based on these results we assigned miR-124-5p and miR-92a-1-5p to be further studied in mouse models, particularly, as they exclusively activate TLR7 and induce TLR7-dependent cytokine release in mouse. miR-9-5p and miR-501-3p were selected to be further studied in human models since both activate hTLR7, and in addition, miR-9-5p activates hTLR8 and induces cytokine release from human macrophages.

**Table 1:** Full mature sequences, previously reported TLR-activating motifs (indicated in italics), and braindead scores of selected MiRNAs. (+/-) indicates whether the miRNA was predicted to activate or not activate the receptor. Respective braindead scores are shown in brackets, and a score of 0.65 was set as the threshold for activation

miRNA	Mature sequence	BrainDead prediction	
		mTLR7	hTLR8
mmu-miR-124-5p	CG*UGUUC*ACAGCGGACCUUGAU	+ (0.717)	+ (0.651)
mmu-miR-92a-1-5p	AGGUUGGGAU*UUGU*CGCAAUGCU	+ (0.905)	+ (0.695)
hsa-miR-9-5p	UCUUUGG*UUAUC*UAGCUGUAUGA	+ (0.925)	+ (0.731)
hsa-miR-501-3p	AAUGCACCCGGGCAAGGAUUCU	- (0.098)	- (0.331)

### Cortical neurons endocytose extracellular miR-124-5p and miR-92a-1-5p

**Fig. 2:**
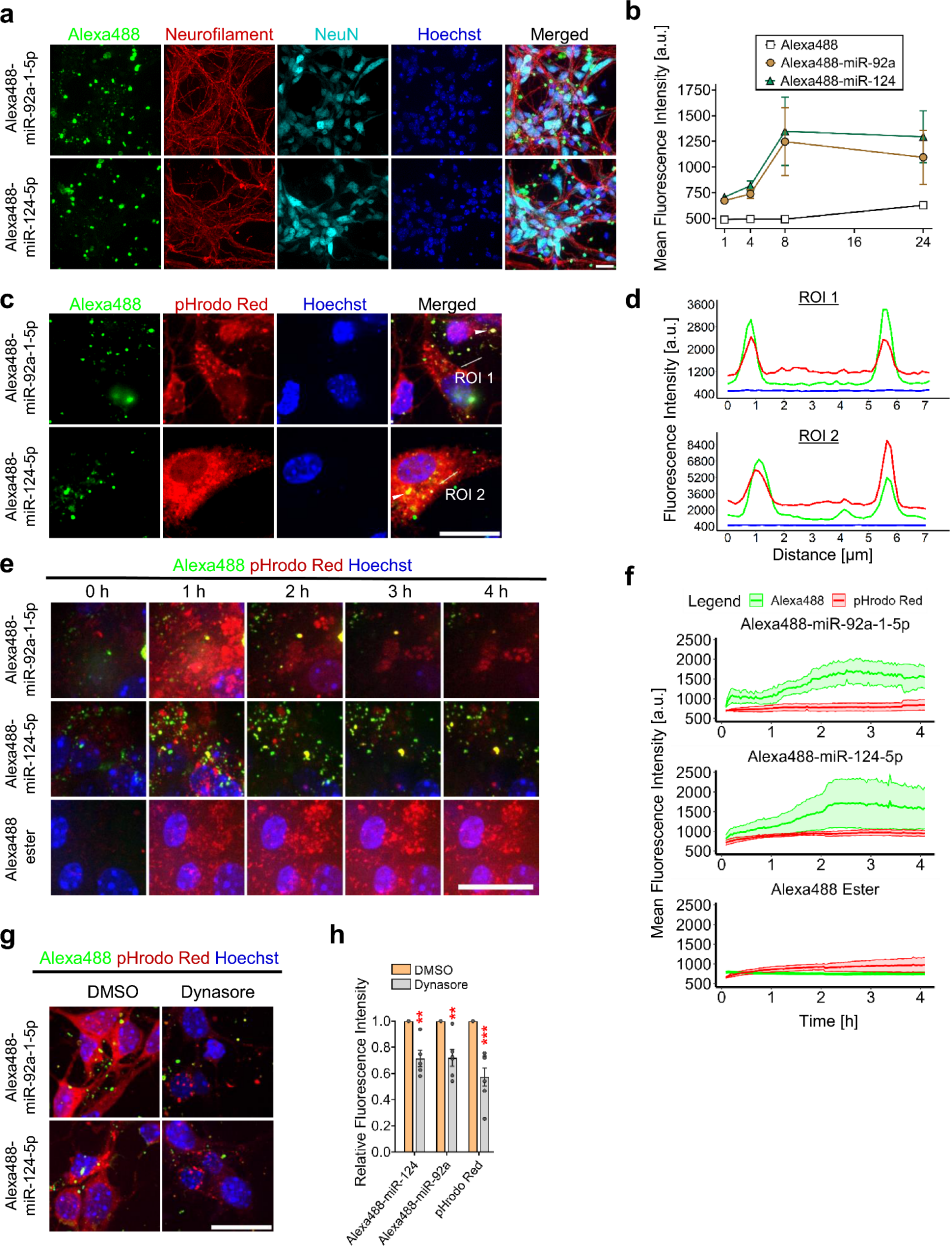
Extracellular miR-124-5p and miR-92a-1-5p enter neurons and co-localize to endosomes. (**a**) C57BL/6 cortical neurons were incubated with Alexa488-labeled 10 µg/mL of miR-92a-1-5p, Alexa488-labeled miR-124-5p (both), or unconjugated Alexa488 ester. Cells stained with Hoechst and immunolabeled with Neurofilament or NeuN antibodies were analyzed by confocal microscopy. Representative images of neurons exposed to miRNAs for 8 h. (**b**) Mean fluorescence intensity of Alexa-488-labeled miRNAs and ester within neurons, depicted as time course graph. Lines and error bars represent mean ± SEM (*n* = 3–4). (**c**) Representative images of neurons incubated with pHrodo Red (20 µg/mL) and fluorescent miRNAs (10 µg/mL), as indicated, for 4 h. Cells were stained with Hoechst and analyzed by confocal microscopy with sequential analysis. Arrow heads indicate fluorescent miRNAs co-localising to pHrodo Red Dextran. White lines indicate regions of interest (ROI). (**d**) Line profiles depicting fluorescence intensities along the marked ROIs. (**e**) Representative images of neurons treated as described in (**c**), set up for confocal time-lapse imaging for 4 h. (**f**) Kinetic curves depicting the fluorescence intensity of fluorescent miRNAs or ester and pHrodo Red within endosomes over the 4 h imaging period. (**g**) Neurons described above treated in parallel with DMSO or Dynasore (200 µM) for 4 h and analyzed by confocal microscopy. (**h**) Fluorescence intensity of fluorescent miRNAs and pHrodo Red within endosomes of neurons described above. Bars represent mean ± SEM (*n* = 5). **P < 0.01; ***P < 0.001, unpaired *t*-test, compared to the corresponding DMSO group. Scale bar, 20 μm

Neurons express ssRNA-sensing TLRs in endosomes [4, 5, 20]. To test whether AD-associated miRNAs identified as TLR7/8 ligands above are taken up by neurons, primary murine cortical neurons were incubated with Alexa488-labeled miR-92a-1-5p or miR-124-5p, both of which preferentially activate mTLR7 (see Fig. 1). Subsequent analysis by confocal microscopy revealed the presence of fluorescent miR-92a-1-5p and miR-124-5p within neurons, mostly around the nuclei, but also in axons (Fig. 2a). Fluorescence intensity of the uptaken miRNA increased over time and reached a peak at 8 h (Fig. 2b). In contrast, the fluorescence intensity of unconjugated Alexa488 ester remained constantly low through the whole observation period (Fig. 2b). To test whether miRNAs taken up by neurons reach the endosomal compartment, murine cortical neurons were incubated with Alexa488-labelled miR-124-5p or miR-92a-1-5p, and pHrodo Red Dextran, an endosomal marker, in parallel. Subsequent confocal microscopy analysis revealed distinct puncta of the fluorescent miRNAs co-localizing to pHrodo Red Dextran, indicating the internalization of the miRNAs by endosomes (Fig. 2c). Sequential analysis of the line profiles from the confocal images confirmed co-localization of the miRNAs to neuronal endosomes (Fig. 2d).

To analyze the kinetics of the extracellular miRNAs’ uptake by neurons, we performed confocal time-lapse imaging of cortical neurons exposed to fluorescent miR-124-5p or miR-92a-1-5p, and pHrodo Red Dextran. Both miRNAs co-localized to pHrodo Red Dextran as early as 20 min after exposure start (Fig. 2e; see Additional files 2 and 3 for movies). Subsequently, fluorescence intensity of endosomal Alexa488 and pHrodo Red was quantified over time (Fig. 2f). The resulting kinetic curves were similar for neurons incubated with Alexa488-labelled miR-124-5p or miR-92a-1-5p, showing a steady increase in the Alexa488 fluorescence intensity within the endosomes. Fluorescence intensity of both miRNAs peaked around 2.5 h (Fig. 2f). In contrast, fluorescence intensity of the unconjugated Alexa488 ester remained at baseline throughout the recording session (Fig. 2f; see Additional file 4 for movie), indicating that it did not accumulate. Likewise, in all time-lapse imaging experiments, the fluorescence intensity of pHrodo Red Dextran within the endosomes remained unchanged (Fig. 2f; see Additional files 2–4 for movies).

Extracellular oligonucleotides are internalized by cells through various mechanisms, including endocytosis [45–48]. The GTPase dynamin is required for vesicle scission from clathrin-coated pits, a key step in clathrin-dependent endocytosis [49]. To assess whether dynamin-mediated endocytosis is involved in the neuronal uptake of miR-124-5p and miR-92a-1-5p, cortical neurons were exposed to Alexa488-labeled miR-124-5p or miR-92a-1-5p with and without Dynasore, a non-competitive small molecule dynamin inhibitor [50]. In neurons treated with Dynasore, fluorescence intensity of the internalized Alexa488-labelled miRNAs within the endosomes was reduced. Similarly, the pHrodo Red Dextran fluorescence intensity was diminished in Dynasore-treated neurons (Fig. 2g, h).

Taken together, extracellular miR-124-5p and miR-92a-1-5p are taken up by neurons via dynamin-dependent endocytosis and accumulate within endosomes.

**Fig. 3:**
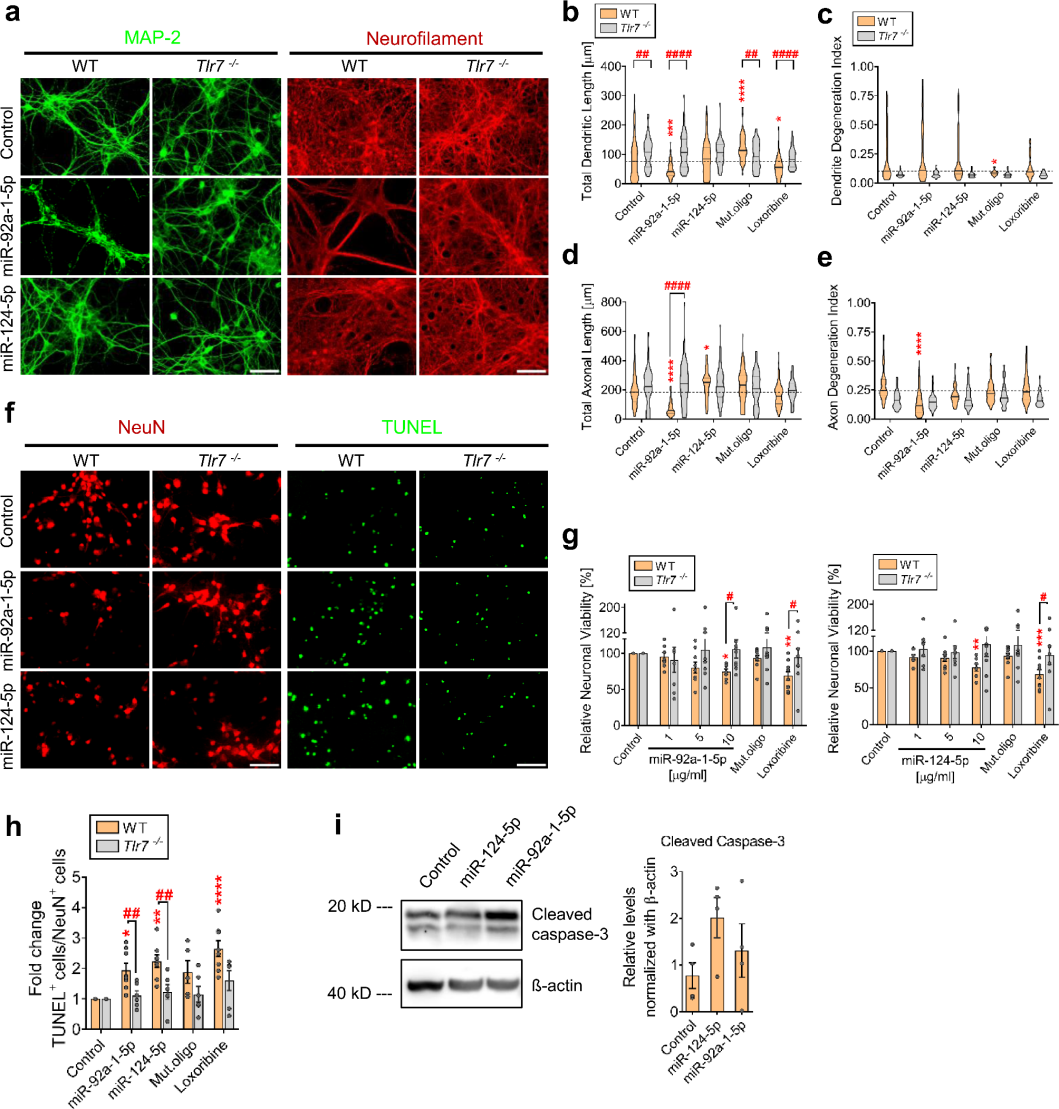
Extracellular miR-124-5p and miR-92a-1-5p alter dendritic and axonal outgrowth and induce neuronal apoptosis. (**a**) Representative images of C57BL/6 (WT) and *Tlr7*^*−/−*^ cortical neurons incubated with 10 µg/mL miR-92a-1-5p or miR-124-5p for 5 d and subsequently, immunolabeled with MAP-2 and Neurofilament antibodies. Untreated cells served as negative control. Quantification of (**b**) dendritic length, (**c**) dendritic degeneration index, (**d**) axonal length, and (**e**) axonal degeneration index of WT and *Tlr7*^*−/−*^ neurons treated as described above. Mut.oligo (10 µg/mL) served as sequence specificity control, while loxoribine (1 mM) served as TLR7 activation control. Violin plot: solid line indicates median, horizontal dotted line indicates the median of the WT control group (*n* = 4–6). *P < 0.05; **P < 0.01; ***P < 0.001; ****P < 0.0001, compared to respective control, Kruskal-Wallis test with Dunn’s post-hoc analysis. ^##^*P* < 0.01; ^####^*P* < 0.0001, compared to corresponding *Tlr7*^*−/−*^ group, Mann-Whitney test. (**f**) Representative images of WT and *Tlr7*^*−/−*^ cortical neurons treated as described above, immunolabeled with NeuN antibody and stained with TUNEL assay. Quantification of (**g**) neuronal viability and (**h**) TUNEL-positive cells, normalized to respective controls. Bars represent mean ± SEM (*n* = 7–9). *P < 0.05; **P < 0.01; ***P < 0.001, compared to respective control, Kruskal-Wallis test with Dunn’s post-hoc analysis. ^#^*P* < 0.05; ^##^*P* < 0.01; compared to corresponding *Tlr7*^*−/−*^ group, Mann-Whitney test. (**i**) Immunoblot depicting cleaved caspase-3 expression in WT neurons exposed to 10 µg/mL miR-92a-1-5p or miR-124-5p for 5 d. β-actin served as loading control (left). Quantification of cleaved caspase-3 expression in neurons, normalized to β-actin (right). Bars represent mean ± SEM (*n* = 3–4). Scale bar, 50 μm

### Exposure to miR-124-5p and miR-92a-1-5p leads to altered structure and viability of CNS neurons

Apart from neuronal loss, dendritic, axonal, and synapse abnormalities in AD have been described [1, 2]. To assess the AD-associated miRNAs’ effects on neurite structure, murine cortical neurons were exposed to miR-124-5p and miR-92a-1-5p, and dendrites and axons were subsequently analyzed by immunocytochemistry (Fig. 3a). Dendritic length of neurons treated with miR-92a-1-5p was reduced to a similar level as observed in loxoribine-treated neurons (Fig. 3a, b). In contrast, dendritic length increased after exposure to Mut.oligo (Fig. 3b). However, no alteration of the dendritic length after exposure to miR-124-5p was detected (Fig. 3a, b), indicating miRNA sequence-specific effects. miR-92a-1-5p-, loxoribine-, and Mut.oligo-induced effects on dendrites required TLR7, as neurons isolated from *Tlr7*^*−/−*^ mice were not affected by the respective treatment (Fig. 3b). To test whether the miR-92a-1-5p-induced reduction in dendritic length was due to fragmentation, i.e. injury, we computed a degeneration index, defined as the ratio of the total area of neurite fragments to the total neurite area [26]. Neither miR-92a-1-5p nor loxoribine treatment increased the dendritic degeneration index (Fig. 3c). In contrast, wild-type (WT) neurons, but not *Tlr7*^*−/−*^ neurons, treated with Mut.oligo exhibited a reduced dendritic degeneration index (Fig. 3c). Of note, native *Tlr7*^*−/−*^ neurons exhibited an increased dendritic length and reduced dendritic degeneration index compared to untreated WT neurons (Fig. 3b, c). Overall, these results indicate that the miR-92a-1-5p-induced reduction of the dendritic length was not due to injury, but rather an effect of restricted outgrowth.

Next, we assessed the effect of extracellular miR-124-5p and miR-92a-1-5p on axons. Axonal length of cortical neurons exposed to miR-92a-1-5p was reduced (Fig. 3d). Conversely, miR-124-5p treatment increased axonal length (Fig. 3d). Neither Mut.oligo nor loxoribine treatment affected axonal length (Fig. 3d). In *Tlr7*^*−/−*^ neurons, miR-92a-1-5p- and miR-124-5p treatment did not affect axonal length, indicating a crucial role for TLR7 herein (Fig. 3d). Assessment of the degeneration index revealed reduced fragmentation of WT axons exposed to miR-92a-1-5p (Fig. 3e), indicating that the miRNA-induced reduction of axonal length was not likely due to injury, but rather an effect of restricted axonal outgrowth.

Certain extracellular miRNAs, such as *let-7b*, induce neuronal cell death [4, 20]. To determine whether extracellular miR-92a-1-5p and miR-124-5p affect neuronal survival, relative neuronal viability of murine cortical neurons exposed to miR-92a-1-5p and miR-124-5p was assessed (Fig. 3f). We observed a dose-dependent decrease in neuronal numbers after exposure to both miRNAs (Fig. 3f, g). In comparison with *let-7b* and loxoribine, miR-92a-1-5p and miR-124-5p induced similar effects, with a reduction in cell number by 35%, 31%, 26%, and 23%, respectively (Additional file 1: Fig. S1). *Tlr7*^*−/−*^ neurons were protected against miR-124-5p- and miR-92a-1-5p-induced neurotoxicity (Fig. 3g). Numbers of apoptotic cells in WT neuronal cultures exposed to miR-124-5p or miR-92a-1-5p were increased, as assessed by TUNEL assay (Fig. 3f, h). In contrast, the apoptosis rate of *Tlr7*^*−/−*^ neurons was unchanged compared to control (Fig. 3h). Expression of cleaved caspase-3 in WT neurons exposed to miR-124-5p and, although to a lesser extent, miR-92a-1-5p was increased (Fig. 3i).

**Fig. 4:**
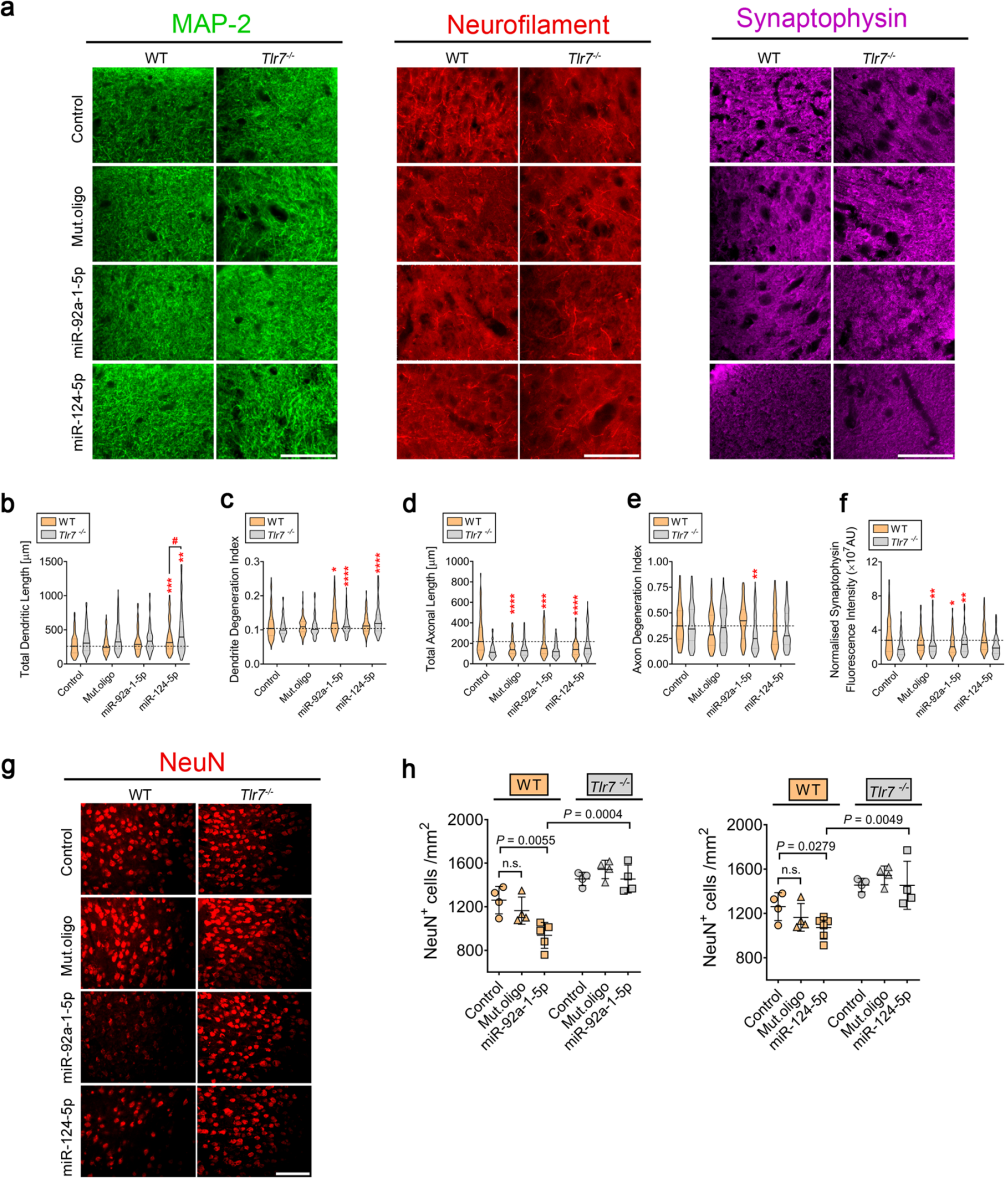
Intrathecal miR-124-5p and miR-92a-1-5p trigger changes in dendrite and axon structure, synaptophysin expression, and cell viability in the mouse cerebral cortex via TLR7. (**a**) Representative images of brain cortical sections from C57BL/6 (WT) or *Tlr7*^*−/−*^ mice. Each mouse received intrathecal injection of either 10 µg miR-92a-1-5p, miR-124-5p, or Mut.oligo. After 3 d, mice were sacrificed, and brain sections were immunolabeled with MAP-2, Neurofilament, and Synaptophysin antibodies. Quantification of (**b**) dendritic length, (**c**) dendrite degeneration index, (**d**) axonal length, (**e**) axonal degeneration index, and (**f**) synaptophysin expression in the brain sections described above. Violin plot: solid line indicates median, horizontal dotted line indicates the median of the WT control group (*n* = 4–6). *P < 0.05; **P < 0.01; ***P < 0.001; ****P < 0.0001, compared to respective control, Kruskal-Wallis test with Dunn’s post-hoc analysis. ^##^*P* < 0.01 compared to corresponding *Tlr7*^*−/−*^ group, Mann-Whitney test. (**g**) Representative images of the brain sections described above, immunolabeled with NeuN antibody. (**h**) Quantification of NeuN^+^ cells in brain sections described above. Bars represent mean ± SEM (*n* = 4–6 per indicated condition), unpaired *t*-test. n.s., not significant. Scale bar, 50 μm

To evaluate the effects of miR-124-5p and miR-92a-1-5p acting as signaling molecules for CNS neurons in vivo, both WT and *Tlr7*^*−/−*^ mice were intrathecally injected with miR-124-5p, miR-92a-1-5p, or Mut.oligo. Immunohistochemical analysis of the cerebral cortex 3 d after injection revealed an increase in dendritic length of cortical neurons in miR-124-5p-treated WT mice (Fig. 4a, b), while miR-92a-1-5p and Mut.oligo did not induce such changes (Fig. 4a, b), indicating miRNA sequence-specific effects on dendrites in vivo. The effect of miR-124-5p on dendritic length did not require TLR7, as cortical neurons of *Tlr7*^*−/−*^ mice showed a similar response (Fig. 4a, b). miR-124-5p did not affect the dendritic degeneration index in WT mice, however, it increased the index in *Tlr7*^*−/−*^ mice. Notably, miR-92a-1-5p increased the dendritic degeneration index in cortical neurons of both WT and *Tlr7*^*−/−*^ mice (Fig. 4c).

Next, the effect of miR-124-5p and miR-92a-1-5p on axons in vivo was assessed. Neurons in the WT cerebral cortex exhibited reduced axonal length after miR-92a-1-5p, miR-124-5p, and Mut.oligo application (Fig. 4a, d). All of these effects were abolished in *Tlr7*^*−/−*^ neurons, indicating a key role for TLR7 in the miRNA-induced effects on axons (Fig. 4a, d). Whereas miR-124-5p and Mut.oligo did not affect the axonal degeneration index, miR-92a-1-5p decreased the index in *Tlr7*^*−/−*^, but not WT neurons (Fig. 4e). These results suggest that the oligoribonucleotide-induced effects on WT axons were due to restricted outgrowth in vivo.

We then examined the effect of the intrathecally applied miRNAs on synapses. Synaptophysin expression in the cerebral cortex of miR-92a-1-5p-treated WT mice was reduced. miR-124-5p or Mut.oligo did not induce such an effect (Fig. 4a, f). In *Tlr7*^*−/−*^ cortices synaptophysin expression was increased by both miR-92a-1-5p and Mut.oligo compared to control (Fig. 4a, f).

Finally, the effect of intrathecal miR-124-5p and miR-92a-1-5p on cortical neuronal viability was analyzed (Fig. 4g). Both miR-124-5p and miR-92a-1-5p induced neuronal loss in WT mice, whereas Mut.oligo had no effect on neuronal numbers (Fig. 4g, h). In contrast, *Tlr7*^*−/−*^ mice were completely protected from neuronal loss induced by miR-124-5p and miR-92a-1-5p (Fig. 4g, h).

Taken together, whereas miR-92a-1-5p exposure resulted in reduced dendritic and axonal length, miR-124-5p enhanced axonal, but not dendritic length in vitro. Both miRNAs in extracellular form induced neuronal apoptosis via caspase-3. Extracellular introduction of miR-92a-1-5p and miR-124-5p into the CSF of mice led to alterations of dendritic and axonal length, synapse protein expression, and resulted in neuronal apoptosis in the cerebral cortex. Most of the miRNA-induced effects required TLR7. A summary of the effects induced by the extracellularly delivered miRNAs in the murine CNS is given in Additional file 1: Table S2.

**Fig. 5:**
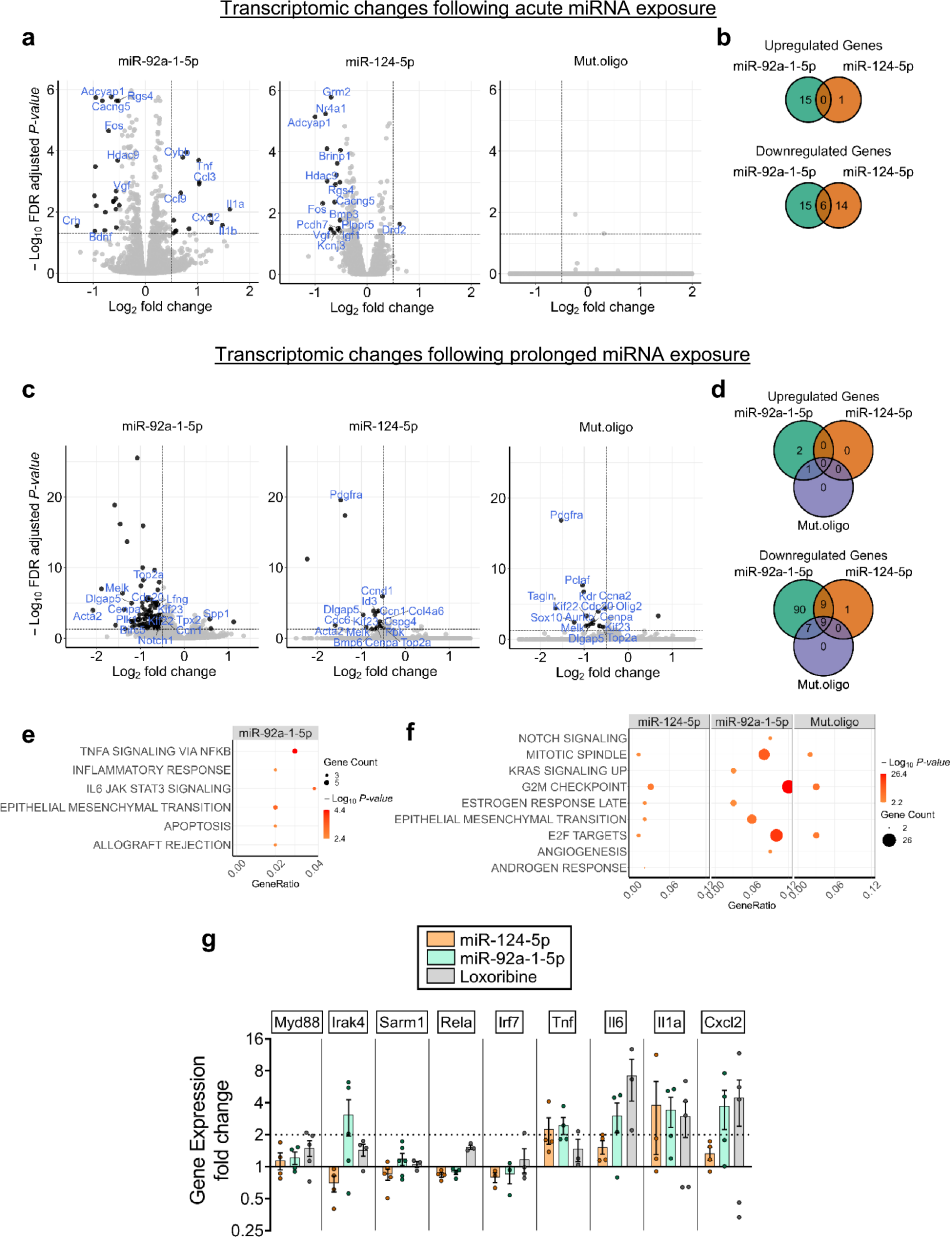
Extracellularly applied miR-124-5p and miR-92a-1-5p induce transcriptomic changes in murine cortical neurons. C57BL/6 (WT) cortical neurons were incubated with 10 µg/mL miR-92a-1-5p, miR-124-5p, or Mut.oligo. After 6 h (**a**,** b**) or 5 d (**c**,** d**), total RNA was harvested from cells, sequenced, and transcriptomic analysis was performed. Volcano plots showing significantly deregulated genes after (**a**) 6 h and (**c**) 5 d (black dots, adjusted *P*-value < 0.05, Log_2_Fold Change > 0.5). Selected genes are labeled. Venn diagrams depicting the intersection of deregulated genes in WT neurons after (**b**) 6 h and (**d**) 5 d exposure to indicated miRNAs. Numbers indicate gene counts for each set. Dot plots showing deregulated pathways in neurons after (**e**) 6 h and (**f**) 5 d exposure to indicated miRNAs. Color intensity and dot size correspond to the *-*Log_10_*P*-value and number of deregulated genes, respectively. (**g**) WT neurons were incubated with miR-92a-1-5p, miR-124-5p (both 10 µg/mL), or loxoribine (1 mM) for 6 h. Expression of select genes, as indicated, was analyzed by qRT-PCR with actin serving as reference gene. Bars represent mean ± SEM of gene expression fold changes, normalized to control (*n* = 3–6)

### Exposure of cortical neurons to miR-124-5p and miR-92a-1-5p leads to changes in inflammation-, proliferation-, and apoptosis-related gene expression

To determine the transcriptome of CNS neurons exposed to AD-associated miRNAs, RNA-seq of murine cortical neurons incubated with miR-92a-1-5p, miR-124-5p, or Mut.oligo for 6 h or 5 d was performed. Early gene expression changes in response to miRNA exposure were assessed after 6 h, before apoptosis would occur (see Fig. 3). Later transcriptional changes, i.e. after miRNA-induced modification of neuronal structure and apoptosis have been started (see Fig. 3), were assessed after 5 d. Setting a cut-off at FDR < 0.05, transcriptome analysis after 6 h miRNA exposure revealed 21 genes downregulated by miR-92a-1-5p, and 20 genes downregulated by miR-124-5p treatment (Fig. 5a, b). In contrast, 15 genes were upregulated by miR-92a-1-5p, while one gene was significantly upregulated by miR-124-5p treatment (Fig. 5a, b). After 6 h, no genes were deregulated by Mut.oligo (Fig. 5a). Six genes were downregulated by both miR-92a-1-5p and miR-124-5p treatment (Fig. 5b, Additional file 1: Table S3). As both miR-92a-1-5p and miR-124-5p activate mTLR7 and induce neuronal apoptosis (see Figs. 1 and 3), we compared the genes deregulated in cortical neurons by exposure to miR-92a-1-5p and miR-124-5p with genes previously described as deregulated after TLR7 activation [51]. Similarly to the TLR7 agonist CL075 [51], miR-92a-1-5p treatment upregulated *Il1a*,* Cxcl2*,* Ccl3*,* Ccl9*, and *Tnf* expression (Fig. 5a), while miR-124-5p downregulated *Grm2* expression (Fig. 5a). We then analyzed all 51 genes deregulated in response to miR-124-5p and miR-92a-1-5p for concordant and discordant expression by performing a disco analysis across all 3 oligoribonucleotide treatments. The disco score is considered proportional to the log-fold change and *P*-value of the gene expression changes in two experimental conditions and exhibits a negative value if the expression changes are in opposite directions [52]. All deregulated genes were concordantly modulated (i.e. disco score > 0). There was a high correlation of expression fold changes for the comparisons miR-92a-1-5p vs. Mut.oligo (*R*^*2*^ > 0.92; Additional file 1: Fig. S2a), miR-124-5p vs. Mut.oligo (*R*^*2*^ > 0.93; Additional file 1: Fig. S2b), and miR-92a-1-5p vs. miR-124-5p (*R*^*2*^ > 0.84; Additional file 1: Fig. S2c), indicating that exposure of neurons to these miRNAs for 6 h triggers a common pattern of gene expression changes.

Transcriptome analysis of cortical neurons exposed to miR-92a-1-5p, miR-124-5p, or Mut.oligo for 5 d revealed 115, 19, and 16 genes as being downregulated, respectively (Fig. 5c, d). Only a few upregulated genes were detected (3 genes after miR-92a-1-5p and one gene after Mut.oligo exposure; Fig. 5c, d). We then wondered whether miR-92a-1-5p, miR-124-5p, or Mut.oligo regulate any genes in common. One gene was upregulated by both miR-92a-1-5p and Mut.oligo (Fig. 5d, Additional file 1: Table S3). In contrast, 9 genes were downregulated by all 3 oligoribonucleotide treatments, another 9 genes were downregulated by miR-92a-1-5p and miR-124-5p, and 7 genes were downregulated by miR-92a-1-5p and Mut.oligo (Fig. 5d, Additional file 1: Table S3). Our disco analysis herein revealed that most of the deregulated genes (97.5%) were concordantly modulated by all 3 oligoribonucleotide treatments. There was a high positive correlation between the expression fold changes: miR-92a-1-5p vs. Mut.oligo (*R*^*2*^ > 0.83; Additional file 1: Fig. S2d), miR-124-5p vs. Mut.oligo (*R*^*2*^ > 0.86; Additional file 1: Fig. S2e), and miR-92a-1-5p vs. miR-124-5p (*R*^*2*^ > 0.77; Additional file 1: Fig. S2f). Thus, 5 d exposure of neurons to the oligoribonucleotides modulated gene expression in a similar fashion.

Next, we performed gene ontology (GO) enrichment analysis of the deregulated genes using the MsigDB Hallmark and GO Biological Process (GO: BP) gene set collections. From the Hallmark collection, “TNF signaling via NfKB”, “IL6 JAK STAT3 signaling”, and “apoptosis” were among the terms overrepresented among genes deregulated after 6 h miR-92a-1-5p exposure (Fig. 5e). Our analysis did not identify any overrepresented terms after exposure to miR-124-5p for 6 h. From the GO: BP collection, “cell-cell signaling”, “synaptic signaling”, and “regulation of transmembrane transport” were among the 15 terms overrepresented among genes deregulated after exposure to both miR-92a-1-5p and miR-124-5p for 6 h (Additional file 1: Fig. S3a, Additional file 5: Table S4). GO enrichment analysis of the deregulated genes in neurons exposed to the oligoribonucleotides for 5 d revealed that categories in the Hallmark collection related to cell proliferation such as “G2M checkpoint”, “E2F targets”, and “mitotic spindle” were overrepresented among genes deregulated by miR-92a-1-5p, miR-124-5p, and Mut.oligo treatment (Fig. 5f). Similarly, in the GO: BP collection “cell cycle”, “cell division”, and “regulation of cell cycle” were enriched among genes deregulated by miR-92a-1-5p, miR-124-5p, and Mut.oligo exposure for 5 d (Additional file 1: Fig. S3b, Additional file 5: Table S4).

The originally established miRNA function, that is regulation of gene expression by post-transcriptional repression of target mRNAs, is particularly exploited and discussed in the context of RNA interference studies and therapeutic strategies, in which extracellular small interfering RNAs silence the expression of target genes in cells [53]. To assess the role of such miRNA-induced mechanisms in cortical neurons exposed to miR-92a-1-5p and miR-124-5p, we performed gene set enrichment analyses (GSEA) of all deregulated genes from our transcriptomic analysis using defined target genes for miR-92a-1-5p and miR-124-5p from several publicly available datasets, namely miRTarBase (with experimentally validated miRNA targets), the MSigDB microRNA targets gene set collection, and TargetScanMouse. None of the miRNA target gene sets were significantly enriched among the deregulated genes identified above (Table 2), indicating that the effects of the tested, extracellularly delivered miRNAs on cortical neurons are not likely mediated via their known gene targets.

**Table 2:** Enrichment scores (E) and adjusted *P*-values (*Q*-values) of published target gene sets among genes D.regulated in C57BL/6 cortical neurons exposed to miR-124-5p and miR-92a-1-5p (both 10 µg/mL) for 6 h or 5 D

miRNA	Database	Number of genes in the gene set	6 h exposure			5 d exposure		
			Number of deregulated genes in the gene set	E	*Q*-value	Number of deregulated genes in the gene set	E	*Q*-value
miR-92a-1-5p	Misgdb_mir	40	0	0	1	0	0	1
	Target Scan	3106	0	0	1	15	0.76	0.91
	miRTarBase	60	0	0	1	1	2.62	0.64
miR-124-5p	Misgdb_mir	103	0	0	1	0	0	1
	Target Scan	3632	0	0	1	6	1.61	0.30
	miRTarBase	52	0	0	1	0	0	1

### Extracellular miR-92a-1-5p and miR-124-5p differently induce expression of inflammatory TLR pathway elements in CNS neurons and microglia

To assess the effect of extracellular miR-124-5p and miR-92a-1-5p, as well as TLR7 activation on the neuronal expression of TLR signaling pathway elements and downstream proinflammatory molecules, murine cortical neurons were incubated with miR-124-5p, miR-92a-1-5p, or loxoribine for 6 h and subsequently, were analyzed by RT-qPCR (Fig. 5g). Whereas exposure to miR-92a-1-5p resulted in increased *Irak4*, *Tnf*,* Il6*, *Il1a*, and *Cxcl2* expression, miR-124-5p treatment led to an increased *Tnf* and *Il1a* expression only. Loxoribine increased neuronal *Il6*, *Il1a*, and *Cxcl2* expression (Fig. 5g). For the experiments outlined above we used highly enriched murine neuron cultures whose substantial contamination with glia is frequently ruled out [4]. To confirm the absence of microglia that would potentially contribute to the observed gene expression changes in neurons exposed to miRNA, and to compare both cell populations regarding their response to miRNAs acting as signaling molecules, both enriched neuronal and microglial cultures were incubated with miR-124-5p or miR-92a-1-5p (Additional file 1: Fig. S4a, b). Loxoribine served as positive control for TLR7 activation, while lipopolysaccharide (LPS) was used as a potent activator of microglia via TLR4 [24] (Additional file 1: Fig. S4a, b). Incubation of microglia with miR-92a-1-5p resulted in downregulation of *Irak4* expression, while this gene was upregulated in miR-92a-1-5p-treated neurons (Fig. 5g). Similarly to neurons, microglia responded to miR-92a-1-5p with increased *Tnf* and *Il6* expression (Additional file 1: Fig. S4a). Exposure of microglia to miR-124-5p led to increased *Irf7*, *Tnf*, and *Il6* expression (Additional file 1: Fig. S4a), while neurons responded with an increase in *Tnf* and *Il1a* expression only (Fig. 5g), confirming our RNAseq data (see Fig. 5a). Loxoribine induced an increase in *Rela*, *Tnf*, and *Il6* expression in microglia (Additional file 1: Fig. S4a), while in neurons, *Il6*, *Il1a*, and *Cxcl* expression was increased (Fig. 5g). While LPS induced increased *Irf7*, *Tnf*, and *Il6* expression in microglia, expression of these genes was decreased in neurons. *Rela* expression in microglia and neurons was not affected by LPS (Additional file 1: Fig. S4b). Overall, these data indicate that the observed gene expression changes in neurons exposed to miR-124-5p and miR-92a-1-5p are not attributable to the presence of microglia.

### Extracellular miR-9-5p and miR-501-3p induce changes in structure and viability of human neurons in a sequence-dependent fashion through TLR7 and TLR8

**Fig. 6:**
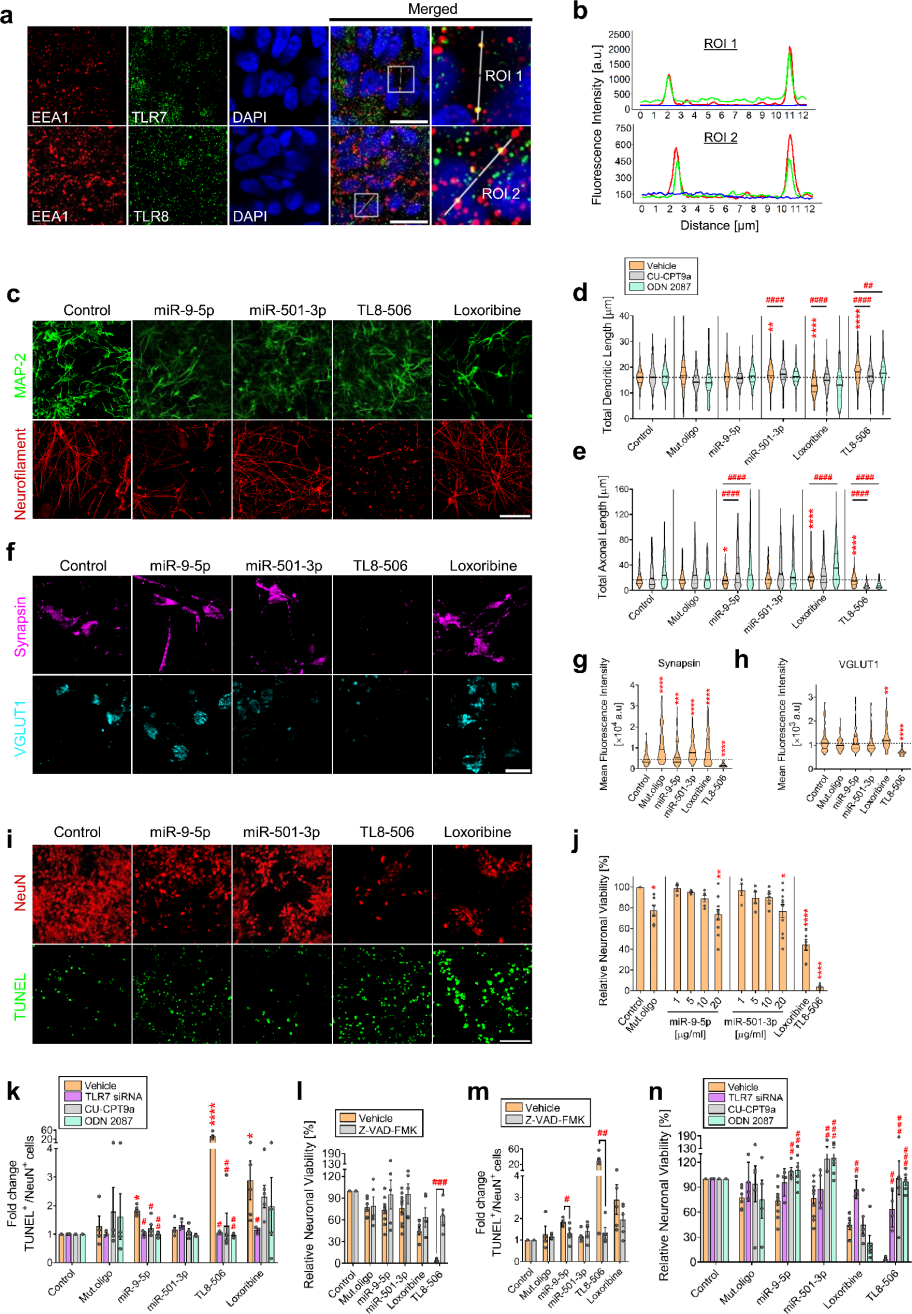
Human neurons exposed to miR-9-5p and miR-501-3p reveal alterations of dendrites, axons, synapse protein expression, and cell viability dependent on sequence and TLR7/8. (**a**) Representative images of iPSC-derived human cortical neurons (iNeurons) marked with DAPI and EEA1 and TLR7 or TLR8 antibodies. White lines indicate regions of interest (ROI). Scale bar, 20 μm. (**b**) Line profiles showing fluorescence intensities along the marked ROIs in iNeurons. (**c**) iNeurons were incubated with 20 µg/mL miR-9-5p or miR-501-3p, loxoribine (1 mM), or TL8-506 (10 µg/mL). After 4 d, cells were immunolabelled with MAP-2 and Neurofilament antibodies. Scale bar, 100 μm. Quantification of (**d**) dendritic length and (**e**) axonal length of iNeurons treated as described above, in the presence of CU-CPT9a (50 µM,) or ODN2087 (10 µM). Violin plot: solid line indicates median, horizontal dotted line indicates the median of the vehicle control group (*n* = 3–6). (**f**) iNeurons treated as described above were immunolabelled with Synapsin and VGLUT1 antibodies. Scale bar, 20 μm. Quantification of (**g**) Synapsin and (**h**) VGLUT1 expression in iNeurons described in (**c**). Violin plot: solid line indicates median (*n* = 3). (**i**) iNeurons treated as described in (**c**) were immunolabelled with NeuN antibody and stained with TUNEL assay. Scale bar, 100 μm. (**j**) Quantification of viability of iNeurons treated as described above. Bars represent mean ± SEM (*n* = 4–7). (**k**) Quantification of TUNEL-positive iNeurons treated as described above in the presence of CU-CPT9a (50 µM), ODN2087 (10 µM), or TLR7 siRNA (0.25 µM). Bars represent mean ± SEM (*n* = 4–7). Quantification of (l) iNeuron viability and (**m**) apoptotic iNeurons treated as described above in the presence of Z-VAD-FMK (100 µM). Bars represent mean ± SEM (*n* = 4–7). (**n**) Quantification of iNeuron viability after exposure to miRNAs for 4 d as described in (**c**) in the presence of CU-CPT9a, ODN2087, or TLR7 siRNA. Bars represent mean ± SEM (*n* = 4–7). *P < 0.05; **P < 0.01; ***P < 0.001; ****P < 0.0001, compared to respective control, Kruskal-Wallis test with Dunn’s post-hoc analysis. ^#^*P* < 0.05; ^##^*P* < 0.01; ^###^*P* < 0.001; ^####^*P* < 0.0001, compared to corresponding vehicle group, Mann-Whitney test

To investigate the response of human CNS neurons to extracellular miRNAs dysregulated in AD, we employed miR-9-5p and miR-501-3p, which preferentially activate hTLR7/8 (see Fig. 1). Both miRNAs are expressed in the human brain and are deregulated in AD [41–43, 54]. For our studies in human, we used cultures of induced pluripotent stem cell (iPSC)-derived human neurons (termed as iNeurons thereafter), using a protocol, which produces predominantly excitatory cortical neurons [22, 55]. First, we tested whether TLR7 and TLR8 are expressed in iNeurons. Both TLR7 and TLR8 protein was readily detectable in the endosomes of iNeurons (Fig. 6a, b). Also, cerebral organoids derived from iNeurons expressed TLR7 and TLR8 protein (Additional file 1: Fig. S5). Next, iNeurons were incubated with miR-9-5p, miR-501-3p, and Mut.oligo for 4 d. Loxoribine and TL8-506 were used as positive controls for TLR7 and TLR8 activation, respectively (Fig. 6c). Fluorescence microscopy analysis revealed an increase in the dendritic length of miR-501-3p- and TL8-506-treated iNeurons. In contrast, dendrite length was reduced by loxoribine. Neither miR-9-5p nor Mut.oligo affected dendritic length (Fig. 6c, d). Axonal length of iNeurons was reduced by miR-9-5p and TL8-506 treatment but was increased by loxoribine (Fig. 6c, e). Neither miR-501-3p nor Mut.oligo induced such effects (Fig. 6c, e). miR-9-5p, Mut.oligo, and TL8-506 treatment reduced the dendritic degeneration index of iNeurons, while no such effect was seen in cells exposed to miR-501-3p or loxoribine (Additional file 1: Fig. S6a). Both miR-9-5p and TL8-506 increased the axonal degeneration index of iNeurons, while loxoribine decreased it (Additional file 1: Fig. S6b), suggesting that the reduction in axonal length induced by miR-9-5p and TL8-506 was due to injury. Also, our data indicate that the modulation of dendrites and axons of iNeurons depends not only on the miRNA’s sequence but also on the involved RNA-sensing receptors.

To further analyze the role of TLR7 and TLR8 in the miRNA-induced changes of neurite structure, we used the TLR8-specific antagonist CU-CPT9a and the dual TLR7/8 antagonist ODN2087. Pre-incubation of iNeurons with CU-CPT9a and ODN2087 abolished the effect of TL8-506 on dendritic length (Fig. 6d). However, ODN2087 treatment did not alter the effect of miR-501-3p or loxoribine on dendrites (Fig. 6d). CU-CPT9a enhanced the miR-501-3p-induced effect on dendrites and conversely, abolished the effect of loxoribine (Fig. 6d). Both CU-CPT9a and ODN2087 abolished the effect of miR-9-5p on axonal length (Fig. 6e). Unexpectedly, ODN2087 potentiated the respective effects of TL8-506 and loxoribine on axons (Fig. 6e). Likewise, CU-CPT9a enhanced the response to TL8-506 (Fig. 6e). Thus, extracellular miR-501-3p and miR-9-5p modulate dendrites and axons of iNeurons, respectively, through TLR7 and TLR8, and the receptors seem to interact herein.

To determine the effects of extracellular miRNA on human synapses, iNeurons were incubated with miR-9-5p, miR-501-3p, or Mut.oligo for 4 d, after which synapses were immunolabeled with antibodies against synapsin, a general marker for synapses, and VGLUT1, a specific marker for excitatory synapses (Fig. 6f). Fluorescence microscopy showed that miR-9-5p, miR-501-3p, and Mut.oligo, increased synapsin expression in iNeurons to a similar extent as loxoribine, while TL8-506 reduced synapsin expression (Fig. 6f, g). In contrast to the TLR agonists, neither the tested miRNAs nor Mut.oligo affected VGLUT1 expression (Fig. 6h).

To test whether the viability of human CNS neurons is affected by extracellular miRNA, iNeurons were exposed to various concentrations of miR-9-5p and miR-501-3p, as well as Mut.oligo. We observed a dose-dependent decrease in neuronal viability in response to miR-9-5p and miR-501-3p (Fig. 6i, j). At the highest concentration applied (20 µg/mL) Mut.oligo also reduced neuronal viability. Oligoribonucleotide-induced neurotoxicity was less pronounced compared to the one induced by loxoribine and TL8-506 (Fig. 6i, j). miR-9-5p and, although not reaching statistical significance, miR-501-3p and Mut.oligo exposure increased apoptosis in iNeuron cultures (Fig. 6k). Preincubation with the broad-spectrum caspase inhibitor Z-VAD-FMK completely protected iNeurons against miR-501-3p- and miR-9-5p-induced cell death, restored neuronal viability, and to some extent also protected iNeurons against loxoribine- and TL8-506-induced neurotoxicity (Fig. 6l). Caspase inhibition had no influence on Mut.oligo-induced neuronal injury (Fig. 6l). Also, caspase inhibition reduced numbers of TUNEL-positive cells exposed to miR-9-5p and TL8-506 (Fig. 6m). In contrast, numbers of apoptotic iNeurons exposed to miR-501-3p and Mut.oligo were not affected by caspase inhibition (Fig. 6m).

To determine the role of TLR7 and/or TLR8 in miRNA-induced neuronal cell death, we again employed CU-CPT9a and ODN2087. Also, we knocked down TLR7 in iNeurons using TLR7 short interfering RNA (siRNA). Pre-incubation with the TLR7/8 antagonists or knock down of TLR7 completely protected iNeurons from miR-9-5p- and miR-501-3p-induced neurotoxicity (Fig. 6n). While loxoribine-induced neurotoxicity was not affected by CU-CPT9a, TLR7 knockdown abolished loxoribine-induced neurotoxicity, and surprisingly, also TL8-506-induced neurotoxicity, but did not affect the neurotoxic effects of miR-9-5p, miR-501-3p, and Mut.oligo (Fig. 6n). Unexpectedly, ODN2087 did not affect loxoribine-induced neurotoxicity, and the reason for this lack of effect remained unclarified. ODN2087, CU-CPT9a, and TLR7 knockdown completely abolished miR-9-5p-induced neuronal TUNEL-positivity (Fig. 6k), indicating an involvement of both TLR7 and TLR8 in the injurious response to miR-9-5p. ODN2087, CU-CPT9a, and TL8-506 abolished TL8-506-induced apoptosis, and TLR7 knockdown, but not CU-CPT9a, protected from loxoribine-induced TUNEL-positivity (Fig. 6k).

Taken together, extracellular miR-9-5p and miR-501-3p modulate human axonal and dendritic length, affect synapse protein expression, and induce neurotoxicity. Also, our data point to an intricate interplay between TLR7 and TLR8 in human neurons modulated by extracellular miRNA. A summary of the effects induced by the extracellularly delivered miRNAs on human iNeurons is given in Additional file 1: Table S5.

## Discussion

Here, we show that the AD-associated miRNAs miR-92a-1-5p, miR-124-5p, miR-9-5p, and miR-501-3p act as extracellularly active signaling molecules in the CNS, thereby modulating structure and viability of cortical neurons. Most of the observed miRNA-induced effects on neurons required TLR7/8 signaling. Indeed, we identified the miRNAs named above as novel agonists of TLR7 and/or TLR8. Aside from miR-501-3p, the above-mentioned miRNAs contain AU-rich and/or GU-rich sequence motifs required for hTLR7/8 activation [56]. However, miR-501-3p lacking such TLR recognition motifs, was capable of hTLR7 activation, suggesting that this miRNA contains yet unknown TLR7-activating sequences. TLR activation by the extracellular miRNAs was dependent on species, as miR-92a-1-5p and miR-124-5p preferentially activated mTLR7, miR-501-3p exclusively activated hTLR7, and miR-9-5p, whose sequence is conserved in both species, elicited a response from hTLR7/8, but not mTLR7/8. Exploring the effects of these miRNAs identified as endogenous TLR7/8 ligands on CNS neurons, we observed that cortical neurons, which express both receptors [4, 57, 58], readily take up extracellular miR-124-5p and miR-92a-1-5p. In contrast to microglia and macrophages, whose response to extracellular miRNA requires complexation with cationic lipids [4, 6, 20], cortical neurons internalized miRNA without transfection. In line with this, the uptake of naked oligoribonucleotides, termed gymnosis [59, 60], primarily by cell lines, but also primary neurons has been reported [61, 62]. Upon neuronal uptake, miR-124-5p and miR-92a-1-5p localized to endosomes, allowing the miRNAs access to the RNA-detecting TLRs located herein. Given that two miRNAs, each comprising different sequences, rapidly entered the neuronal endosomal compartment, this saturable process does not seem to depend on a specific longer RNA sequence. Nonetheless, future studies investigating further miRNAs are needed to determine whether miRNAs contain certain sequence and/or structure motifs that are required for their uptake by neurons. Also, as the dynamin-inhibitor employed in our study impairs fluid-phase endocytosis independent of dynamin [63], further studies may validate the role of dynamin-mediated miRNA endocytosis by CNS neurons and evaluate further mechanisms of their RNA uptake, such as caveolin-dependent endocytosis and micropinocytosis.

In AD, dendritic, axonal, as well as synapse abnormalities contribute to malfunctioning of the brain and may be forerunners to neuronal loss [1, 2]. Based on our previous [4, 20] and current data we suggest a mechanism whereby injured neurons release miRNAs, which - being stably present in the extracellular space - can act as signaling molecules on neighboring neurons, thereby triggering neurodegeneration. miR-92a-1-5p and miR-124-5p, whose expression is downregulated in apoptotic neurons [20, 33], in their extracellular form induced injury of cortical neurons via TLR7 and caspase-3. This is in line with previous studies investigating other miRNAs, such as *let-7b* [4, 20], and point to cell injury as a central effect of miRNAs acting as signaling molecules in the CNS. Yet, the extracellular miRNA-triggered signaling elements, the outcome and its extent of the neuronal response may depend on the specific miRNA sequence. Indeed, intrathecal miR-92a-1-5p and miR-124-5p induced different effects in the mouse cerebral cortex. Whereas miR-92a-1-5p led to reduction in dendritic and axonal length and synaptophysin expression, miR-124-5p increased axonal length, without detectable effects on dendrites or synapses. Also, the extent of neuronal loss differed. In human iNeuron cultures, extracellular miR-501-3p increased the dendrite length without effects on axons, while miR-9-5p induced opposite effects. Yet, both miR-9-5p and miR-501-3p increased human synapsin expression. In line with our findings, other miRNAs such as *let-7c* and miR-21 have been reported to restrict dendritic outgrowth [64]. Of note, the extracellular control mutant oligoribonucleotide also affected neurons in our study, e.g. by inducing synapsin expression and cell death in iNeurons, and by inducing gene expression changes in murine neurons after prolonged exposure. These data suggest that not only miRNAs with a specific sequence, but also small RNA molecules independently of their sequence can serve as signalling molecules inducing different transcriptomic changes and cellular responses.

In our study, most of the miRNA-induced effects on both murine and human neurons required TLR7 and/or TLR8. Accordingly, the dual TLR7/8 agonist resiquimod was reported to inhibit neurite outgrowth [65, 66]. We have demonstrated in previous work that miR-100-5p and miR-298-5p, which directly activate TLR7/8, localize to TLR7 and endosomes in murine microglia [20]. Using microscale thermophoresis we confirmed that such miRNAs bind to human TLR8 [20]. In our current study the tested miRNAs directly activated TLR7/8, localized to endosomes after neuronal uptake, and triggered effects on neurons dependent on TLR7/8 function, indicating that these miRNAs act as ligands for these receptors. We suggest that the different responses of cortical neurons to certain miRNAs, sometimes evolving in opposite direction (e.g. decrease vs. increase in neurite length), may be due to an intricate interplay between the specific sequence of the miRNA involved and the respective TLR. Also, in a pathophysiological setting, the outcome of the exposure of neurons to extracellular miRNA may depend on specific subsets of miRNAs, which, individually or together within a pattern, interact with TLR7/8. The complexity of such interactions, potentially resulting in a finetuned modulation of CNS neuronal structure and viability is underlined by a recent study, in which dendritic outgrowth is regulated by both TLR7 and certain cytokines, such as IL-6 [65]. We found that exposure to miR-92a-1-5p, which decreased neurite length, led to upregulation of *Il6* expression in cortical neurons, whereas miR-124-5p, which failed to affect dendrites, did not. Furthermore, exposure to miR-92a-1-5p resulted in downregulation of neuronal *Tpx2* expression, whose depletion reduced neurite length by interfering with microtubules [67]. However, although our study focuses on miRNAs whose expression is altered in AD, our data cannot provide a direct causal link between the miRNA-induced effects on cortical neurons and pathogenic mechanisms involved in AD. While most of the observed miRNA-induced effects were in accordance with degenerative hallmarks in AD, i.e. decrease in neurite length, enhanced neuronal apoptosis, some effects had reverse character, i.e. increase in axonal length and synaptic protein expression. Also, we observed that one miRNA such as miR-124-5p induces an increase in axonal length, while triggering neuronal cell death at the same time. However, while miR-124-5p-induced neuronal cell death required TLR7, the effect on axonal length was independent of TLR7, indicating that extracellular miR-124-5p modulates neuronal viability and axonal length through different mechanisms. Aside from direct TLR activation miRNAs can serve further unconventional functions, such as regulation of ion channel currents [68, 69]. Thus, miR-124-5p might serve as a ligand not only for TLR7 but also for further, yet unidentified receptors, thereby inducing multiple, different signaling pathways, potentially resulting in complex cellular responses. Considering pathogenesis and progression of neurodegenerative diseases including AD as complex processes, our data indicate that miRNAs acting as signaling molecules can modulate cortical neuronal structure and survival, thereby potentially contributing to the disease.

Although we detected TLR7/8 expression in both human iNeurons and human cortical organoids, re-analysis of published transcriptomics data sets of iNeurons generated by similar protocols [70, 71] failed to show constitutive receptor expression (data not shown). Furthermore, our studies on human neurons demonstrate that inhibition of TLR7 and TLR8 function and expression did not only affect the miRNA-induced effects through the respective receptor, but also the other miRNA-sensing TLR. Also, unexpectedly, exposure of neurons to an established TLR8 antagonist and a dual TLR7/8 antagonist enhanced some of the effects induced by the respective TLR agonists. The reason and underlying mechanisms remain unresolved in our study, but we speculate that these effects may be due to TLR redundancy, which is a well-known, but hitherto little understood principle in immune cells [72], and largely unknown in the CNS. Particularly, TLR7 and TLR8 exhibit high redundancy in human [73]. Furthermore, TLR7 and TLR8 expression in cortical neurons may be tightly regulated under yet undefined pathophysiological/pathological conditions, and the miRNA-sensing receptors may closely interact, thereby modulating neuron structure and viability. Further studies are required to resolve such complex interaction between miRNAs and their sensors in CNS neurons in the context of neurodegeneration.

To analyze the transcriptome of neurons challenged by prolonged miRNA exposure, murine cell cultures were incubated with chemically stabilized miRNAs for several days without medium change. Whether the miRNA was still present or degraded to some extent at the end of the experiment remains elusive. However, miRNA is known to be exceptionally stable to degradation in the extracellular space, withstanding even harsh treatments such as numerous freeze-thaw cycles [17, 18]. Based on this stability, miRNAs are discussed as biomarkers for various human diseases including neurodegenerative disease [74, 75]. Thus, we assume that the miRNAs used in our experiments were stable over long time periods. Whether the observed changes on cultured neurons in response to miRNA treatment were due to an initial effect/insult at earlier time points or continuing effects induced by the respective miRNA, is unclear. Although the model of intrathecal injection has been established over the past years in our laboratory to evaluate the effects of extracellular single-stranded RNA in the CNS in vivo [4, 7, 8, 76, 77], the detection of the intrathecally injected miRNA in certain brain regions and different cell types still poses a particular technical challenge, and we cannot rule out that these difficulties are, at least in part, due to degradation of the miRNA after injection. In line with the concept that miRNAs are extracellularly present and active, cerebrospinal fluid of AD patients contains miRNAs, which are released from apoptotic neurons and are capable of neuronal injury [4, 20]. Neurons secrete miRNAs complexed with proteins, such as Argonaute2 and high-density lipoproteins [78–80], and neuronal exosomes contain miRNA subsets, which are distinct from the cellular miRNA profile [81]. Whether miRNAs can be released in non-complexed form, i.e. naked RNA, remains unresolved. While further studies are necessary to elaborate the molecular details of miRNA release from CNS neurons, the principle of an intercellular crosstalk based on extracellular miRNA is well established in human cancer models [19, 82] and may also contribute to human neurodegenerative disease.

We found that exposure of cortical neurons to AD-associated miRNA altered their transcriptomic profiles, particularly with respect to signaling pathways related to inflammation, proliferation, and apoptosis, underlining the miRNAs’ role in the modulation of CNS neurons. While in immune cells TLR7 signals through MyD88, leading to NF-κB and IRF7 activation [5], in cortical neurons *Myd88*, *Rela*, and *Irf7* expression was not affected by miR-124-5p or miR-92a-1-5p treatment, pointing to the involvement of MyD88-independent pathways. Indeed, neuronal apoptosis induced by ssRNA derived from human endogenous retrovirus K, or the TLR7/8 ligand imiquimod requires SARM1, but not MyD88 [7, 83]. Nonetheless, exposure of neurons to miR-92a-1-5p and miR-124-5p resulted in upregulation of *Tnf* and *Il1a*, previously reported to be upregulated after TLR7 activation [51]. As inflammation is a hallmark of neurodegenerative disorders, the observed cytokine response from both neurons and immune cells induced by extracellular AD-associated miRNA may suggest that miRNAs, not only serving as gene regulators, but also acting as signaling molecules, contribute to human neurodegenerative disease. Our RNAseq data did not consistently reflect the miRNA-induced effects on neurons, e.g. alteration of dendrites and axons, observed in the model of intrathecal injection. Several aspects should be considered here: (i) RNAseq data were derived from in vitro approaches comprising only neurons, while the intrathecal miRNA injection represents an in vivo model, in which multiple cell types, e.g. neurons, glia, and the vascular system likely interact in a complex fashion, (ii) different time points of analysis (miRNA exposure over 5 d in vitro vs. 3 d post injection in vivo), and (iii) different developmental stages (embryonic cells in vitro vs. brains of adult mice). Importantly, none of the usual target genes were deregulated in neurons exposed to miR-92a-1-5p and miR-124-5p. Thus, considering that degradation of target mRNA accounts for 66–90% of the miRNA-mediated gene silencing in mammalian cells [84–86], it seems unlikely that the observed responses of neurons to extracellular miRNA were consequences of the miRNA’s canonical gene silencing. Acting as TLR ligands, miRNAs likely induce signalling cascades differing from the ones resulting from the canonical gene silencing. Also, as extracellular miRNAs were observed to enter neuronal endosomes, in order to exert gene silencing effects, miRNAs would need to associate with the RNA-induced silencing complex to target mRNAs [87], which in turn is only possible if endocytosed miRNAs have crossed the endosomal lipid bilayer barrier to enter the cytoplasm, which is considered a major hurdle [88]. Furthermore, uptaken miRNAs accumulating within neuronal endosomes might be degraded over time by nucleases. Considering the foregoing, extracellular miRNAs uptaken by neurons are not likely to escape the endosomal compartment in sufficient amounts allowing them to carry out their canonical gene silencing function.

## Conclusions

Our results show that neurodegenerative disease-associated miRNAs in extracellular form modulate structure and viability of CNS neurons depending on the miRNA’s sequence. Focusing on AD as a representative neurodegenerative disorder our study highlights the potentially complex role of miRNAs acting as signaling molecules in the CNS and may open avenues towards novel therapeutic strategies in neurodegenerative diseases.

## Electronic supplementary material

Below is the link to the electronic supplementary material.

Additional file 1: Supplementary Fig. S1. Extracellular miR-92a-1-5p, miR-124-5p, let-7b, and loxoribine induce neuronal loss. Supplementary Fig. S2. Exposure of neurons to extracellular miR-92a-1-5p and miR-124-5p triggers concordant gene expression. Supplementary Fig. S3. Exposure of neurons to extracellular miR-92a-1-5p and miR-124-5p modulates transcriptional pathways. Supplementary Fig. S4. Extracellular miR-92a-1-5p and miR-124-5p induce inflammatory cytokine expression in microglia. Supplementary Fig. S5. Human cortical organoids express TLR7 and TLR8. Supplementary Fig. S6. Dendritic and axonal degeneration index of iNeurons exposed to miR-9-5p and miR-501-3p. Supplementary Table S1. Primer sequences for qPCR. Supplementary Table S2. Summary of the effects induced by extracellularly delivered miR-92a-1-5p and miR-124-5p in primary neurons and adult mouse brain. Supplementary Table S3. List of genes deregulated in common after 6h and 5d exposure of C57BL/6 cortical neurons to miR-92a-1-5p, miR-124-5p, or Mut.oligo. Supplementary Table S5. Summary of the effects induced by extracellularly delivered miR-9-5p and miR-501-3p in human iNeurons. Supplementary Table S6. Published datasets that were analyzed in this study.

Additional file 2: Live uptake of extracellular Alexa488-labeled miR-124-5p into neuronal endosomes.

Additional file 3: Live uptake of extracellular Alexa488-labeled miR-92a-1-5p into neuronal endosomes.

Additional file 4: Live cell recording showing extracellular Alexa488 ester does not enter neuronal endosomes.

Additional file 5: Supplementary Table S4. Excel file containing complete data for gene set enrichment analysis of genes deregulated in C57BL/6 cortical neurons after 6 h and 5 d exposure to oligoribonucleotides.

## Data Availability

No datasets were generated or analysed during the current study.

## Abbreviations

AD: Alzheimer’s disease
CNS: central nervous system
DMSO: dimethyl sulfoxide
ELISA: enzyme-linked immunosorbent assay
GO: gene ontology
IL: interleukin
LPS: lipopolysaccharide
miRNA: microRNA
NeuN: neuronal nuclei
NF-κB: nuclear factor ‘kappa-light-chain-enhancer’ of activated B-cells SEAP secreted embryonic alkaline phosphatase
ssRNA: single-stranded RNA
TLR: Toll-like receptor
TNF: tumor necrosis factor
TUNEL: TdT-mediated dUTP-biotin nick end labeling
CSF: cerebrospinal fluid
RT-qPCR: real-time quantitative polymerase chain reaction
iPSC: induced pluripotent stem cell
NSC: neural stem cell
PMA: phorbol-12-myristat-13-acetate
