# Supplementary material for: Neurodegenerative disease-associated microRNAs acting as signaling molecules modulate CNS neuron structure and viability
Source: Cell Commun Signal. 2025 Apr 24;23:196. doi: 10.1186/s12964-025-02199-8 (PMC12020182; doi:10.1186/s12964-025-02199-8)
Supplement: Supplementary file 1 — Additional file 1: Supplementary Fig. S1. Extracellular miR-92a-1-5p, miR-124-5p, let-7b, and loxoribine induce neuronal loss. Supplementary Fig. S2. Exposure of neurons to extracellular miR-92a-1-5p and miR-124-5p triggers concordant gene expression. Supplementary Fig. S3. Exposure of neurons to extracellular miR-92a-1-5p and miR-124-5p modulates transcriptional pathways. Supplementary Fig. S4. Extracellular miR-92a-1-5p and miR-124-5p induce inflammatory cytokine expression in microglia. Supplementary Fig. S5. Human cortical organoids express TLR7 and TLR8. Supplementary Fig. S6. Dendritic and axonal degeneration index of iNeurons exposed to miR-9-5p and miR-501-3p. Supplementary Table S1. Primer sequences for qPCR. Supplementary Table S2. Summary of the effects induced by extracellularly delivered miR-92a-1-5p and miR-124-5p in primary neurons and adult mouse brain. Supplementary Table S3. List of genes deregulated in common after 6h and 5d exposure of C57BL/6 cortical neurons to miR-92a-1-5p, miR-124-5p, or Mut.oligo. Supplementary Table S5. Summary of the effects induced by extracellularly delivered miR-9-5p and miR-501-3p in human iNeurons. Supplementary Table S6. Published datasets that were analyzed in this study. [file 12964_2025_2199_MOESM1_ESM.pdf]

Supplementary Figure S1

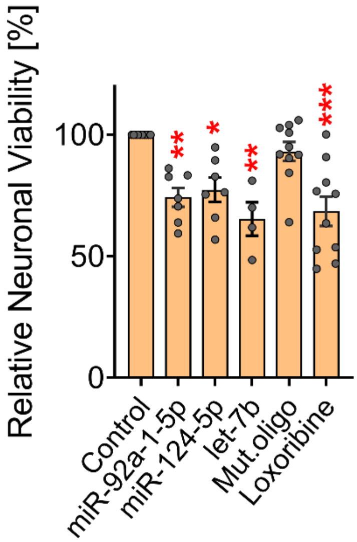

Supplementary Figure S1. Extracellular miR-92a-1-5p, miR-124-5p, *let-7b*, and loxoribine induce neuronal loss. C57BL/6 cortical neurons were incubated with 10 µg/mL of indicated miRNAs or loxoribine (1 mM) for 5 d. Mut.oligo (10 µg/mL) served as sequence specificity control. Subsequently, cells were immunolabeled with NeuN antibody. Quantification of neuronal viability normalized to control group. Bars represent mean ± SEM ($n = 4$-9). *$P < 0.05$; **$P < 0.01$; ***$P < 0.001$, compared to control, Kruskal-Wallis test with Dunn's post-hoc analysis.

Supplementary Figure S2

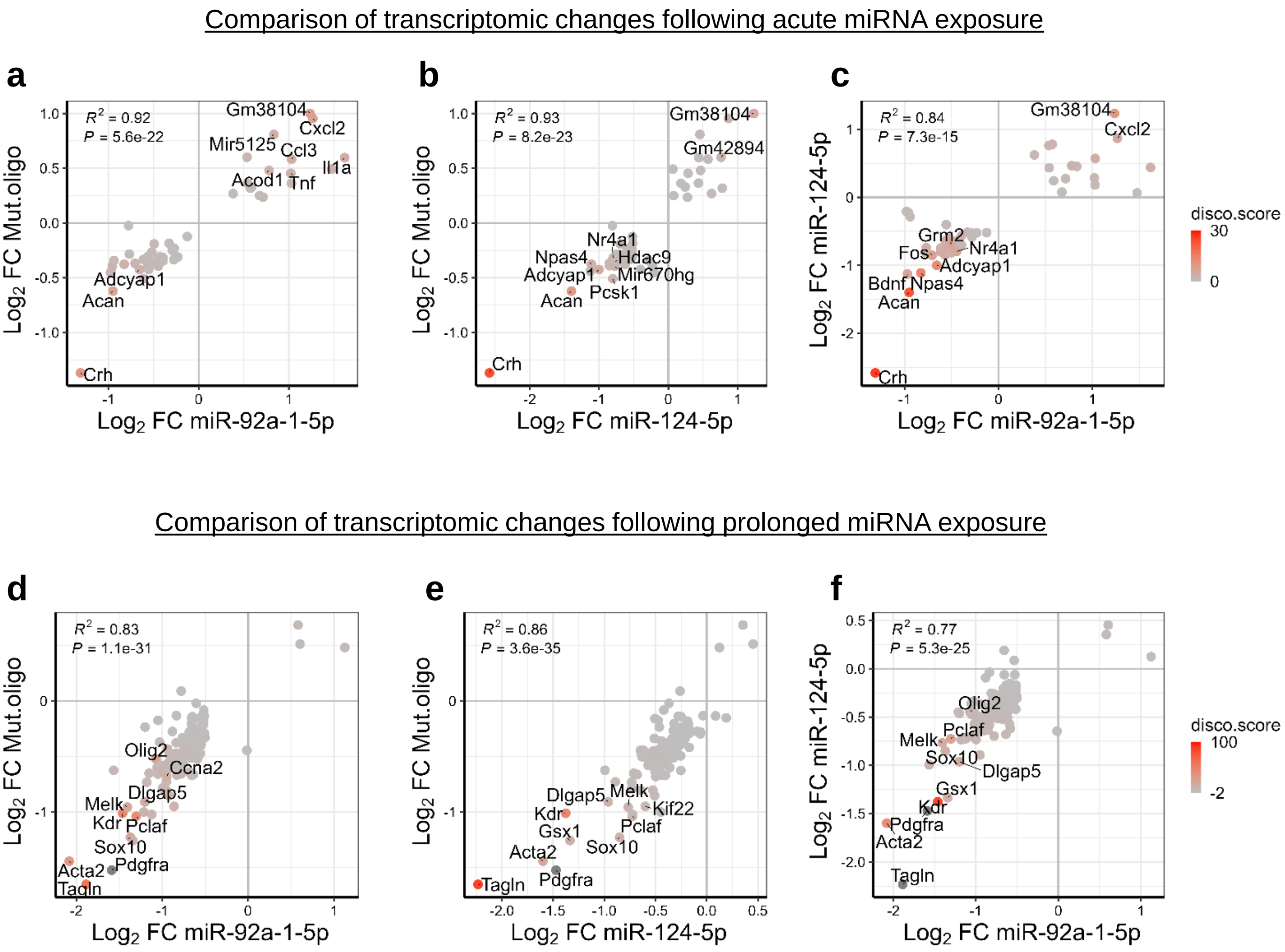

Supplementary Figure S2. Exposure of neurons to extracellular miR-92a-1-5p and miR-124-5p triggers concordant gene expression. Disco plots comparing expression changes of significantly deregulated genes in C57BL/6 cortical neurons after exposure to miR-92a-1-5p, miR-124-5p, or Mut.oligo. Acute exposure (6h): (**a**) Mut.oligo vs miR-92a-1-5p, (**b**) miR-124-5p vs miR-92a-1-5p, (**c**) miR-124-5p vs miR-92a-1-5p. Prolonged exposure (5d): (**d**) Mut.oligo vs miR-92a-1-5p, (**e**) miR-124-5p vs miR-92a-1-5p (**f**) miR-124-5p vs miR-92a-1-5p. The top 10 concordant genes based on disco.score are labeled. The color-coded bar represents the disco.score.

Supplementary Figure S3

a

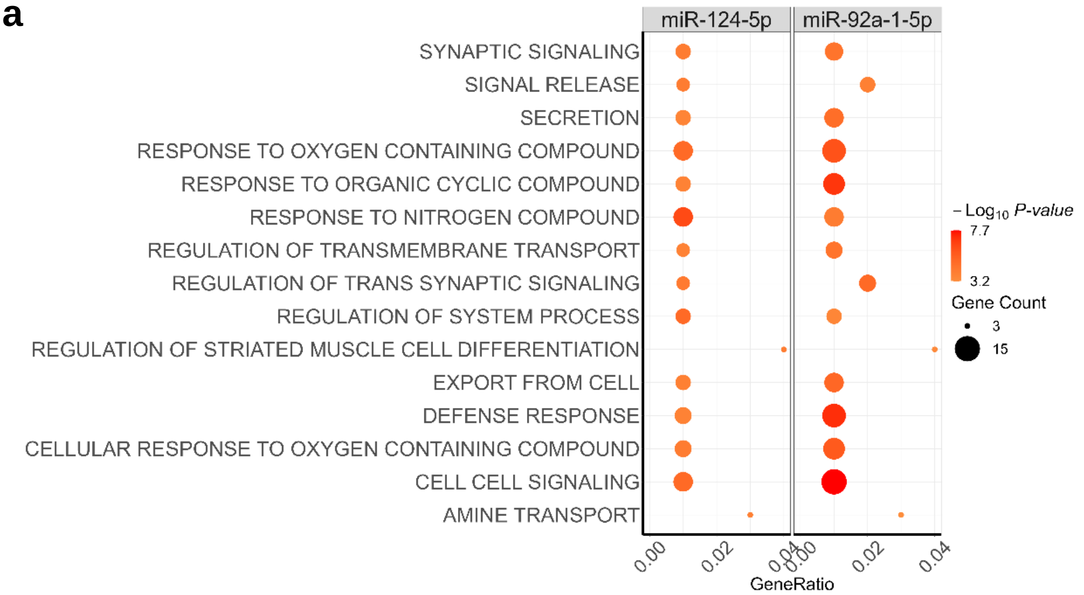

b

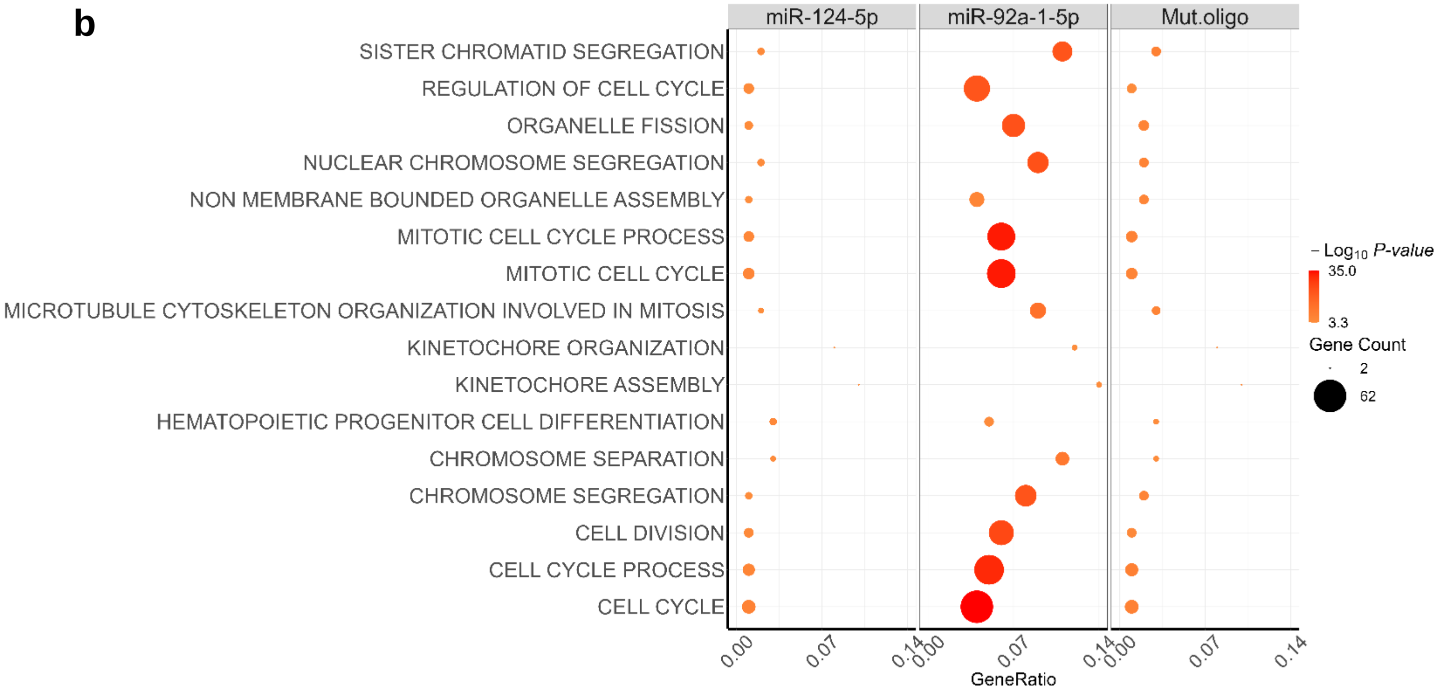

Supplementary Figure S3. Exposure of neurons to extracellular miR-92a-1-5p and miR-124-5p modulates transcriptional pathways. Dot plots showing selected enriched Gene Ontology (GO) terms of deregulated genes in C57BL/6 cortical neurons after (**a**) 6 h and (**b**) 5 d exposure to indicated miRNAs. The color intensity and size of the dots correspond to the $-\text{Log}_{10}$ *P*-value and number of deregulated genes, respectively.

Supplementary Figure S4

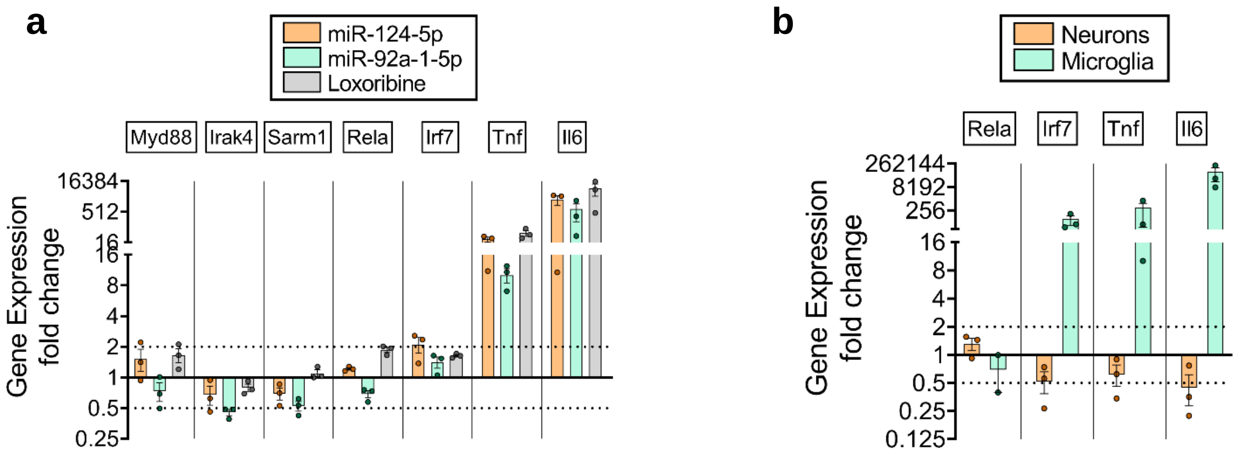

Supplementary Figure S4. Extracellular miR-92a-1-5p and miR-124-5p induce inflammatory cytokine expression in microglia (**a**) Expression of TLR signaling elements and cytokines in C57BL/6 microglia exposed to miR-92a-1-5p, miR-124-5p (both 10 μg/mL), or loxoribine (1 mM) for 6 h, as assessed by qRT-PCR. (**b**) Expression of TLR signaling elements and cytokines in C57BL/6 cortical neurons and microglia exposed to LPS (100 ng/mL) for 6 h. Bars represent mean ± SEM of gene expression fold changes, normalized to control (*n* = 3-5). Actin (*Actb*) served as reference gene.

.

Supplementary Figure S5

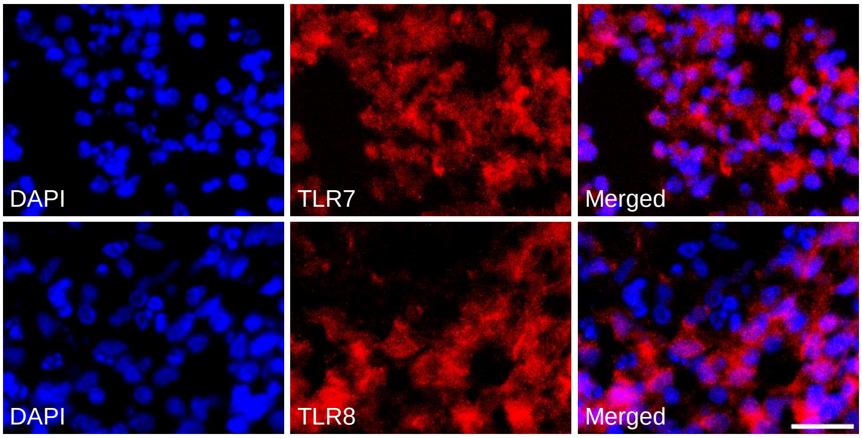

Supplementary Figure S5. Human cortical organoids express TLR7 and TLR8. Representative images of human cortical organoids labeled with antibodies against TLR7 or TLR8. Scale bar, 20 μm.

.

Supplementary Figure S6

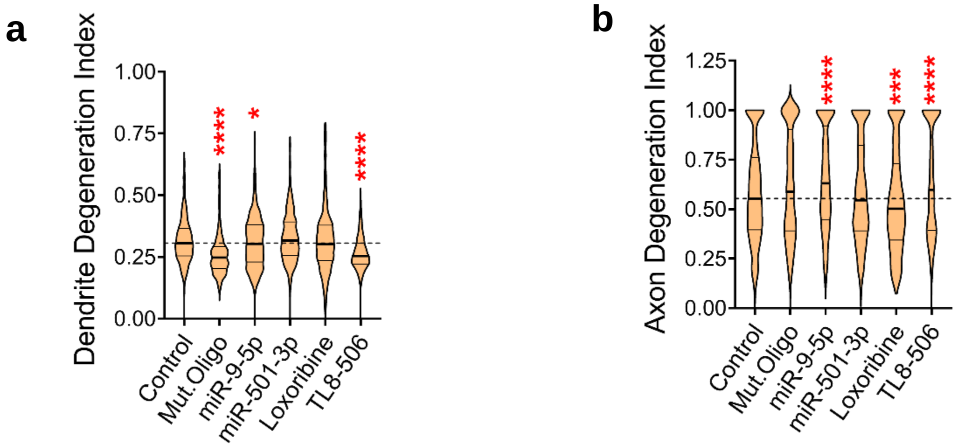

Supplementary Figure S6. Dendritic and axonal degeneration index of iNeurons exposed to miR-9-5p and miR-501-3p. Quantification of (**a**) dendrite and (**b**) axon degeneration index of iNeurons exposed to miR-9-5p, miR-501-3p, Mut.oligo (all 20 µg/mL), loxoribine (1 mM), or TL8-506 (10 µg/mL) for 4 d. Violin plot: solid line indicates median (*n* = 3-6). **P* < 0.05; ****P* < 0.001; *****P* < 0.0001, compared to respective control, Kruskal-Wallis test with Dunn's post-hoc analysis.

.

Supplementary Table S1. Primer sequences for qPCR.

Target Gene	Forward Primer	Reverse Primer
Sarm1	TTCCTTGGCTCCAGAAATGCT	GACCCTGAGTTCCTCCGGTA
Irak4	CATACGCAACCTTAATGTGGGG	GGAACTGATTGTATCTGTCGTCG
Actb	CCTGAACCCTAAGGCCAAC	GACAGCACAGCCTGGATGG
Myd88	ACCTGTGTCTGGTCCATTGCCA	GCTGAGTGCAAACTTGGTCTGG
Rela	TCCTGTTCGAGTCTCCATGCAG	GGTCTCATAGGTCCTTTTGCGC
Irf7	CCTCTGCTTTCTAGTGATGCCG	CGTAAACACGGTCTTGCTCCTG
Tnf	GGTGCCTATGTCTCAGCCTCTT	GCCATAGAACTGATGAGAGGGAG
Il6	TACCACTTCACAAGTCGGAGGC	CTGCAAGTGCATCATCGTTGTTC
Ticam1	ATCCATGCCAGGGCTGATGAAC	CGATGGCATCTTGGAGACAGTG
Il1a	CGAAGACTACAGTTCTGCCATT	GACGTTTCAGAGGTTCTCAGAG
Cxcl2	CCAACCACCAGGCTACAGG	GCGTCACACTCAAGCTCTG

Supplementary Table S2. Summary of the effects induced by extracellularly delivered miR-92a-1-5p and miR-124-5p in primary neurons and adult mouse brain.

	WT		
	miR-92a-1-5p	miR-124-5p	Mut.oligo
Neuronal loss (*in vitro*)	▲	▲	▬
Neuronal apoptosis (*in vitro*)	▲	▲	▬
Dendritic length (*in vitro*)	▼	▬	▲
Axonal length (*in vitro*)	▼	▲	▬
Neuronal loss (*in vivo*)	▲	▲	▬
Dendritic length (*in vivo*)	▬	▲	▬
Axonal length (*in vivo*)	▼	▼	▼
Synaptophysin expression (*in vivo*)	▼	▬	▬

	Tlr7$^{-/-}$		
	miR-92a-1-5p	miR-124-5p	Mut.oligo
Neuronal loss (*in vitro*)	●	●	○
Neuronal apoptosis (*in vitro*)	●	●	○
Dendritic length (*in vitro*)	●	○	●
Axonal length (*in vitro*)	●	○	○
Neuronal loss (*in vivo*)	●	●	○
Dendritic length (*in vivo*)	○	●	○
Axonal length (*in vivo*)	○	○	○
Synaptophysin expression (*in vivo*)	○	○	○

Legend

▲	increased effect compared to untreated group
▬	no effect compared to untreated group
▼	decreased effect compared to untreated group
○	no change to effect in respective WT group
●	diminished effect compared to respective WT group

Supplementary Table S3. List of genes deregulated in common after 6h and 5d exposure of C57BL/6 cortical neurons to miR-92a-1-5p, miR-124-5p, or Mut.oligo (all 10 µg/ml).

**Upregulated genes**		
5 d exposure	miR-92a-1-5p and Mut.oligo	*Spp1*
**Downregulated genes**		
6 h exposure	miR-92a-1-5p and miR-124-5p	*Fos, Adcyap1, Vgf, Cacng5, Hdac9, Rgs4*
5 d exposure	miR-92a-1-5p, miR-124-5p and Mut.oligo	*Top2a, Cenpa, Pdgfra, Tagln, Kif23, Melk, Dlgap5, Pclaf, Kdr*
	miR-92a-1-5p and miR-124-5p	*Cdc6, Pbk, Ccn1, Col4a6, Cspg4, Acta2, Bmp6, Cdk6, Ccnd1*
	miR-92a-1-5p and Mut.oligo	*Uhrf1, Cdc20, Aurkb, Ccna2, Kif22, Sox10, Olig2*

Supplementary Table S5. Summary of the effects induced by extracellularly delivered miR-9-5p and miR-501-3p in human iNeurons.

	Vehicle		
	miR-9-5p	miR-501-3p	Mut.oligo
Neuronal loss	▲⑧	▲⑧	▲∅
Neuronal apoptosis	▲⑧⑦	▬	▬
Dendritic length	▬	▲⑧	▬
Axonal length	▼⑧	▬	▬
Synapsin expression	▲○	▲○	▲○
VGLUT1 expression	▬	▬	▬

	CU-CPT9a		
	miR-9-5p	miR-501-3p	Mut.oligo
Neuronal loss	🔴	🔴	●
Neuronal apoptosis	🔴	●	●
Dendritic length	●	🔴	●
Axonal length	🔴	●	●

	ODN 2087		
	miR-9-5p	miR-501-3p	Mut.oligo
Neuronal loss	🔴	🔴	●
Neuronal apoptosis	🔴	●	●
Dendritic length	●	●	●
Axonal length	🔴	●	●

	TLR7 siRNA		
	miR-9-5p	miR-501-3p	Mut.oligo
Neuronal loss	●	●	●
Neuronal apoptosis	🔴	●	●

	z-VAD-FMK		
	miR-9-5p	miR-501-3p	Mut.oligo
Neuronal loss	●	●	●
Neuronal apoptosis	🔴	●	●

Legend

▲	increased effect compared to untreated group
▬	no effect compared to untreated group
▼	decreased effect compared to untreated group
⑦	effect dependent on TLR7
⑧	effect dependent on TLR8
∅	effect not dependent on TLR7/8
○	dependency on TLR7/8 not tested
●	no change to effect in respective Vehicle group
🔴	diminished effect compared to respective Vehicle group

Supplementary Table S6. Published datasets that were analyzed in this study.

Dataset	Source	Identifier
RNAseq data of mouse cortical neurons after TLR7 activation	Hung et al., 2018	GEO: GSE107199
RNAseq data of control human iPSC-derived neurons	Das et al., 2021	GEO: GSE163161
RNAseq data of control human iPSC-derived neurons	Sagar et al., 2023	https://www.mdpi.com/article/10.3390/cells12151990/s1

References:

Hung Y-F, Chen C-Y, Shih Y-C, Liu H-Y, Huang C-M, Hsueh Y-P. Endosomal TLR3, TLR7, and TLR8 control neuronal morphology through different transcriptional programs. J Cell Biol. 2018;217(8):2727-42.

Das D, Peng X, Lam A-TN, Bader JS, Avramopoulos D. Transcriptome analysis of human induced excitatory neurons supports a strong effect of clozapine on cholesterol biosynthesis. Schizophr Res. 2021;228:324-6.

Sagar R, Azoidis I, Zivko C, Xydia A, Oh ES, Rosenberg PB, et al. Excitatory Neurons Derived from Human-Induced Pluripotent Stem Cells Show Transcriptomic Differences in Alzheimer’s Patients from Controls. Cells. 2023;12(15):1990.
